# Computational modeling of eticyclidine drug adsorption and detection on c_60_ based nanostructures

**DOI:** 10.1038/s41598-026-50875-7

**Published:** 2026-05-19

**Authors:** El-Sayed Khafagy, Amr Selim Abu Lila, Mahboubeh Pishnamazi

**Affiliations:** 1 Department of Pharmaceutics, College of Pharmacy, Prince Sattam bin Abdulaziz University, Al-kharj, 11942 Saudi Arabia; 2 Department of Pharmaceutics, College of Pharmacy, University of Ha’il, Ha’il, 81442 Saudi Arabia; 3 Institute of Research and Development, Duy Tan University, Da Nang, Vietnam; 4 School of Engineering & Technology, Duy Tan University, Da Nang, Vietnam

**Keywords:** Eticyclidine, Absorption, Drug detection, Sensor, DFT, Chemistry, Materials science, Nanoscience and technology

## Abstract

The detection of Eticyclidine (PCE) as a psychotropic drug is difficult due to the limitations of conventional analytical techniques (such as the need for expensive instruments, expert labor, and time-consuming). In this computational study, structural, electronic, and optical properties were evaluated to assess the ability of pristine C_60_ fullerene and its aluminum (AlC_59_) and zinc (ZnC_59_) doped forms as sensors/adsorbers for PCE using density functional theory (DFT), time-dependent DFT (TD-DFT), and quantum theory of atoms in molecules (QTAIM). The calculated structural/electronic properties of pristine C_60_ (bond lengths, energy gaps, and UV spectra) were in excellent agreement with reported experimental data, supporting the validity of the computational approach. The weak/reversible binding interaction energy, recovery times, conductivity and reactivity of pure C_60_ indicate its suitability for repeated use as an electrochemical sensor for PCE. However, AlC_59_ has strong and irreversible binding, indicating that it can be used as an excellent adsorbent for PCE removal. ZnC_59_ is moderately bound to PCE compared to C_60_ and AlC_59_. TD-DFT calculations show a large red shift of the UV-Vis spectra when PCE is absorbed by AlC_59_ and ZnC_59_, which provides evidence of strong optical responses, indicating their potential for use as colorimetric sensors. The results obtained from NCI/RDG, ELF, LOL and QTAIM analyses confirm that metal doping significantly enhances the strength of the interaction with PCE. We believe that the findings of this theoretical study can provide a reliable basis for the experimental development of C_60_-based materials for PCE sensing and adsorption.

## Introduction

Eticyclidine, also known as PCE, is a synthetic dissociative drug belonging to the arylcyclohexylamine class, closely related to phencyclidine and ketamine. It was originally investigated in the mid-20th century for potential use as an anesthetic and analgesic because of its ability to block N-methyl-D-aspartate (NMDA) receptors in the brain, which reduces the perception of pain and produces dissociation between consciousness and sensory input. However, due to severe and unpredictable psychological side effects, PCE was never approved for routine medical use and today has no accepted therapeutic application; its presence is mainly limited to illicit recreational use or laboratory research contexts. The risks associated with PCE are significant and multifaceted. Acutely, it can cause intense dissociation, confusion, agitation, anxiety, hallucinations, paranoia, and psychotic episodes, sometimes resembling schizophrenia, along with impaired judgment and coordination that greatly increase the risk of accidents or injury. Physiological effects may include elevated heart rate and blood pressure, nausea, vomiting, numbness, and at higher doses, respiratory depression, seizures, hyperthermia, or loss of consciousness. Repeated or heavy use carries risks of persistent cognitive impairment, memory and attention deficits, mood disturbances, and long-lasting psychosis, as well as the development of tolerance and psychological dependence. Because dosing is unpredictable and purity varies in illicit markets, the risk of overdose is substantial, especially when combined with other substances such as alcohol, opioids, or benzodiazepines. In addition, PCE is classified as a controlled substance in many countries, so its possession or distribution carries legal risks alongside the serious health dangers^1–3^.

PCE can be identified using several methods, including chemical analysis, immunoassays, and spectroscopy. These methods provide different ways to detect the substance, each with its own strengths and limitations. Chemical analysis, such as gas chromatography-mass spectrometry (GC-MS), enables precise identification of PCE by separating and analyzing its chemical components. Immunoassays, such as enzyme-linked immunosorbent assays (ELISA), use antibodies to detect the presence of a drug based on its unique molecular structure. Spectroscopy, such as infrared (IR) or nuclear magnetic resonance (NMR) spectroscopy, analyzes the molecular vibrations or atomic structure of the substance to determine its composition^4,5^. However, each of these identification methods has notable disadvantages. Chemical analysis typically requires trained personnel and expensive equipment, making it impractical for quick or field use. Immunoassays, while faster, are often less specific and may not differentiate between similar substances, leading to potential false positives or negatives. Spectroscopic techniques also demand a laboratory environment and may require long testing times, further complicating their use for rapid identification. Also, the most important disadvantage of each of these methods is that none can be used in real time on-site. Given these challenges, the need for accessible, quick, and reliable methods to identify PCE has become more urgent than ever before. Recently, electrochemical and colorimetric sensors have gained attention in the field of drug detection due to their potential to offer faster, more accessible, and cost-effective alternatives to traditional methods like chemical analysis and spectroscopy^6,7^.

These sensors are mainly designed and synthesized using carbon nanostructures, such as carbon nanotubes, graphene, and fullerenes. Among these carbon-based structures, fullerenes (especially fullerene C_60_) have garnered significant attention. Fullerene C_60_, with its distinctive spherical structure, offers advantages such as a large surface-to-volume ratio, which enhances its ability to interact with target molecules. Additionally, its π-electron transfer capabilities allow for efficient electron exchange, improving the sensitivity and selectivity of the sensors. Recent research has increasingly emphasized the use of C_60_ in sensor development^8^.

For example, Hadi et al. proposed the use of functionalized C_60_ nanocages as both an adsorbent and a sensor for the detection of methamphetamine^9^. Similarly, Pattanaik et al. investigated the potential of boron and silicon functionalized C_60_ as an advanced sensor for the detection of tyramine and demonstrated its high sensitivity and selectivity^10^. Furthermore, Parlak et al. highlighted the performance of functionalized C_60_ as a sensor for molnupiravir, emphasizing its ability to accurately detect in complex environments^11^. These studies not only demonstrate the utility of C_60_ in sensor technology but also highlight the doping of C_60_ as a promising strategy to improve its sensing properties.

In this work, given the importance of identifying PCE and the sensing capabilities of C_60_, we investigate, for the first time, the interaction of PCE with C_60_ and its doping forms with aluminum (Al) and zinc (Zn). The choice of Al and Zn as dopants is based on their unique chemical properties, which enhance C_60_’s performance in sensor applications. Al, when incorporated into the C_60_ carbon framework, exhibits significant Lewis acidity^12^. This creates electron-deficient sites on the fullerene surface, which are expected to exhibit strong electrophilic reactivity. In contrast, Zn was chosen because of its known tendency to form coordination complexes^13^. Recent work also shows that doping Al and Zn into C_60_ can be a successful strategy to improve its sensing properties. For example, Thajudeen et al. showed that Al- and Zn-doped C_60_ exhibits higher sensitivity to isobutyric acid than pristine C_60_^14^. Asogwa et al. also emphasized that Al- and Zn-doping into C_60_ improves its performance in absorbing gaseous pollutants from the environment^15^.

Nowadays, computational methods have become an essential first step before conducting experimental work, particularly in fields such as drug detection and materials science. Theoretical methods minimize trial-and-error in experimental procedures, which not only accelerates the research process but also significantly reduces the costs associated with experiments. By simulating interactions and properties at the molecular or atomic level, computational techniques provide valuable insights that guide the design and optimization of experiments, leading to more targeted and efficient approaches.

In this work, we used a comprehensive computational approach to study the interaction of PCE with C_60_ and its derivatives. This approach combines several advanced theoretical methods, including density functional theory (DFT), time-dependent density functional theory (TD-DFT), and the quantum theory of atoms in molecules (QTAIM)^16,17^. By leveraging these theoretical methods, we aim to gain a deeper understanding of how PCE interacts with C_60_ and its doped forms. We hope that the insights from this computational study will guide and inform experimental work, offering a more efficient path to developing practical sensors and advancing drug-detection technologies. The results of this work are expected to be instrumental in optimizing sensor materials and facilitating the design of more sensitive, selective, and cost-effective detection methods for PCE.

## Computational details

**Fig. 1:**
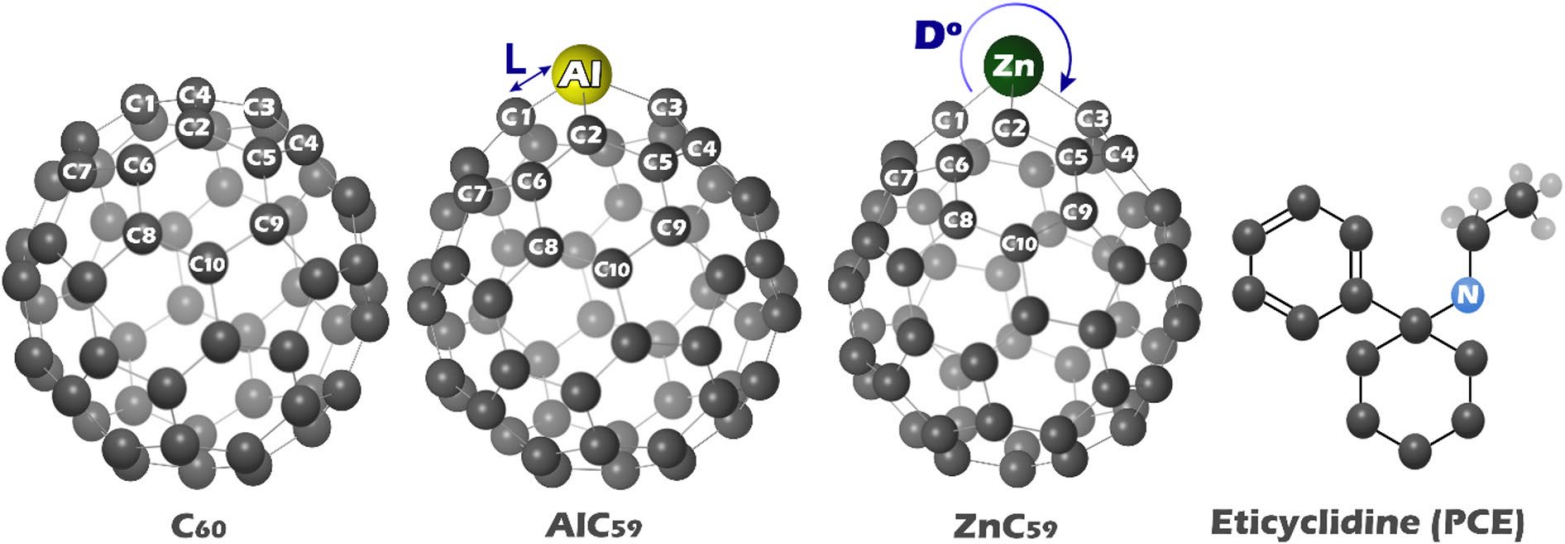
Optimized structure of each of the structures designed in this work.

In this work, the structure of each of the studied structures (C_60_, AlC_59_, ZnC_59_, and PCE) was first drawn using GaussView 6.0 software. Then, their geometry in two isolated states and in complex with PCE (C_60_@PCE, AlC_59_@PCE, ZnC_59_@PCE) was optimized using Gaussian 09 W software^18^. Geometry optimization was performed using density functional theory (DFT), at the B97D/6-31G* computational level (Fig. 1). For future data reproducibility, the XYZ coordinates for all designed structures were reported in Table S1 in the Supplementary Data. In the human body and many biological systems, water is the dominant solvent. Most biochemical reactions, including enzyme activity, drug-receptor interactions, and molecular signaling, occur in an aqueous environment. Therefore, simulation of drug interactions and sensor behavior in water provides a more accurate representation of how these processes occur. For this purpose, the entire optimization process was performed in the water phase using the Conductor-like Polarizable Continuum Model (CPCM)^19^.

The B97D functional was chosen because it accurately describes non-covalent interactions, such as dispersion forces, while maintaining a good balance between computational efficiency and accuracy in systems involving weak interactions^20^. Avramopoulos et al. also showed that the electronic properties calculated for C_60_ (such as the energy gap) using the B97D functional have a significant overlap with the experimental results^21^.

Finally, frequency calculations were also performed at the same theoretical level and no imaginary frequencies were observed, indicating local minima on the potential energy surface, confirming that all structures are stable and not in a transition state.

To investigate the optical properties of the designed systems, time-dependent density functional theory (TD-DFT) calculations were carried out at the same theoretical level used for geometry optimization (B97D/6-31G*). The TD-DFT calculations were performed on the optimized geometries to obtain the vertical excitation energies and corresponding oscillator strengths. The first several singlet excited states were computed in order to determine the maximum absorption wavelength (λmax) and the corresponding excitation energies (Eex). These calculations allow reliable prediction of UV-Vis absorption spectra and provide insight into the electronic transitions responsible for the optical response of the sensor systems.

The cohesive energy ($\:{E}_{Coh}$) for each structure was calculated using Eq. 1.

1 $$\:{E}_{Coh}=\raisebox{1ex}{${E}_{total}-\sum\:{E}_{atom}$}\!\left/\:\!\raisebox{-1ex}{$n$}\right.$$

In this equation, $\:{\mathrm{E}}_{\mathrm{t}\mathrm{o}\mathrm{t}\mathrm{a}\mathrm{l}}$: The total energy of the molecule when it is in its optimized form; $\:\sum\:{E}_{atom}$: The sum of the energies of the individual atoms in the structure if they were isolated; $\:n$: the total number of atoms in the molecule^22^.

The most important reactivity parameters such as energy gap ($$\:\mathrm{H}\mathrm{L}\mathrm{G}=\left|{\mathrm{E}}_{\mathrm{H}\mathrm{O}\mathrm{M}\mathrm{O}}-{\mathrm{E}}_{\mathrm{L}\mathrm{U}\mathrm{M}\mathrm{O}}\right|$$), chemical hardness ($$\:{\upeta\:}=\raisebox{1ex}{$(-{\mathrm{E}}_{\mathrm{H}\mathrm{O}\mathrm{M}\mathrm{O}}-(-{\mathrm{E}}_{\mathrm{L}\mathrm{U}\mathrm{M}\mathrm{O}}\:\left)\right)$}\!\left/\:\!\raisebox{-1ex}{$2$}\right.$$), chemical softness ($$\:S=1/2{\upeta\:}$$), and chemical potential ($$\:{\upmu\:}=-(-{\mathrm{E}}_{\mathrm{H}\mathrm{O}\mathrm{M}\mathrm{O}}+(-{\mathrm{E}}_{\mathrm{L}\mathrm{U}\mathrm{M}\mathrm{O}}\left)\right)/2$$) were studied computationally. $$\:{\mathrm{E}}_{\mathrm{H}\mathrm{O}\mathrm{M}\mathrm{O}}$$ and $$\:{\mathrm{E}}_{\mathrm{L}\mathrm{U}\mathrm{M}\mathrm{O}}$$ represent the energy of the highest occupied molecular orbital and the lowest unoccupied molecular orbital, respectively^23^.

The electronic density of states (DOS) analysis (Using the GaussSum program, version 3.0) was performed to further evaluate the electronic structure and energy distribution of molecular orbitals for the studied systems^24^. DOS spectra were generated from the calculated molecular orbital energies obtained from DFT calculations. These plots illustrate the distribution of electronic states across different energy levels and allow visualization of changes in the HOMO-LUMO gap and electronic structure upon PCE adsorption. DOS analysis therefore provides complementary confirmation of the electronic reactivity trends derived from frontier molecular orbital calculations. Finally, the UV spectrum of each structure was plotted using the GaussSum program, version 3.0. Also, the spatial distribution of frontier molecular orbitals was analyzed to qualitatively evaluate possible charge-transfer pathways between the sensor and the analyte.

The charge-transfer descriptors $\:{\varDelta\:N}_{max}$ and electrophilicity-based charge transfer (ECT) were calculated to evaluate the electron-donating or electron-accepting capability of the studied systems (Eqs. 2 and 3). Here, the parameter $\:{\varDelta\:N}_{max}$ represents the maximum electronic charge transfer capacity predicted by conceptual density functional theory (CDFT) and is calculated from the chemical potential and chemical hardness of the system. It is a dimensionless quantity that estimates the theoretical upper limit of electron exchange between interacting fragments. Importantly, $\:{\varDelta\:N}_{max}$ should not be interpreted as the actual number of electrons physically transferred during complex formation. Instead, it reflects the relative ability of a system to accept or donate electronic charge, which becomes larger for systems with lower chemical hardness (η) and higher electronic softness. In contrast, ECT is a derived descriptor that quantifies the effective charge transfer between the sensor and the analyte by incorporating the electrophilicity difference between the interacting species. Therefore, although ECT is mathematically related to $\:{\varDelta\:N}_{max}$, the two quantities describe different physical aspects: $\:{\varDelta\:N}_{max}$ reflects the intrinsic charge-accepting capacity of an isolated system, whereas ECT reflects the actual direction and magnitude of charge transfer occurring during sensor@analyte interaction based on electrophilicity considerations^25^.

2 $$\:{\varDelta\:\boldsymbol{N}}_{\boldsymbol{m}\boldsymbol{a}\boldsymbol{x}}=-\raisebox{1ex}{$\boldsymbol{\mu\:}$}\!\left/\:\!\raisebox{-1ex}{$\boldsymbol{\eta\:}$}\right.$$

3 $$\:\boldsymbol{E}\boldsymbol{C}\boldsymbol{T}={\left({\boldsymbol{\Delta\:}\boldsymbol{N}}_{\boldsymbol{m}\boldsymbol{a}\boldsymbol{x}}\right)}_{\boldsymbol{\alpha\:}}-{\left({\boldsymbol{\Delta\:}\boldsymbol{N}}_{\boldsymbol{m}\boldsymbol{a}\boldsymbol{x}}\right)}_{\boldsymbol{\beta\:}}$$

In Eq. (3), $\:{\left({\boldsymbol{\Delta\:}\boldsymbol{N}}_{\boldsymbol{m}\boldsymbol{a}\boldsymbol{x}}\right)}_{\boldsymbol{\alpha\:}}$ and $\:{\left({\boldsymbol{\Delta\:}\boldsymbol{N}}_{\boldsymbol{m}\boldsymbol{a}\boldsymbol{x}}\right)}_{\boldsymbol{\beta\:}}$ denote the maximum charge-transfer capacities of the isolated sensor and the Sensor@Drug complex, respectively. Here, the subscripts α and β are not independent parameters but are used solely to distinguish between the two systems under consideration. Specifically, $\:{\left({\boldsymbol{\Delta\:}\boldsymbol{N}}_{\boldsymbol{m}\boldsymbol{a}\boldsymbol{x}}\right)}_{\boldsymbol{\alpha\:}}$ corresponds to the value calculated for the pristine sensor, while $\:{\left({\boldsymbol{\Delta\:}\boldsymbol{N}}_{\boldsymbol{m}\boldsymbol{a}\boldsymbol{x}}\right)}_{\boldsymbol{\beta\:}}$ represents the value obtained for the sensor@analyte complex after interaction. This formulation clarifies that the ECT parameter is derived from the difference in charge-transfer capacities between these two states, reflecting the change in electrophilic behavior upon complex formation.

The adsorption energy ($\:{\mathrm{E}}_{\mathrm{a}\mathrm{d}\mathrm{s}}$), recovery time ($\:\tau\:$), and electrical conductivity ($\:{\upsigma\:}$) were used to investigate the sensing ability (Eqs. 4–6).

4 $$\:{\mathbf{E}}_{\mathbf{a}\mathbf{d}\mathbf{s}}={\mathbf{E}}_{\left(\mathbf{Z}\mathbf{n}/\mathbf{A}\mathbf{l}\right)\mathbf{C}60@\mathbf{D}\mathbf{r}\mathbf{u}\mathbf{g}}-\left({\mathbf{E}}_{\boldsymbol{D}\boldsymbol{r}\boldsymbol{u}\boldsymbol{g}}+{\mathbf{E}}_{\left(\mathbf{Z}\mathbf{n}/\mathbf{A}\mathbf{l}\right)\mathbf{C}60}\right)$$

5 $$\:\boldsymbol{\tau\:}={\boldsymbol{V}}_{0}^{-1}\times\:\mathbf{e}\mathbf{x}\mathbf{p}(-\frac{{\boldsymbol{E}}_{\boldsymbol{a}\boldsymbol{d}\boldsymbol{s}}}{{\boldsymbol{K}}_{\boldsymbol{B}}\boldsymbol{T}})$$

6 $$\:\boldsymbol{\upsigma\:}=\boldsymbol{A}{\boldsymbol{T}}^{3/2}{\boldsymbol{e}}^{(-\boldsymbol{H}\boldsymbol{L}\boldsymbol{G}/2{\boldsymbol{K}}_{\boldsymbol{B}}\boldsymbol{T})}$$

In Eq. 4, $\:{\mathrm{E}}_{\left(\mathrm{Z}\mathrm{n}/\mathrm{B}\right)\mathrm{C}60@\mathrm{D}\mathrm{r}\mathrm{u}\mathrm{g}}$ is the energy of complex formation of each of the sensors (C_60_, AlC_59_, and ZnC_59_) with PCE. $\:{\mathrm{E}}_{Drug}$ and $\:{\mathrm{E}}_{\left(\mathrm{Z}\mathrm{n}/\mathrm{A}\mathrm{l}\right)\mathrm{C}60}$ represent the energy of PCE and each of the sensors in the isolated state, respectively. Equations 5 and 6 define $\:{V}_{0}$ the effort frequency (typically ~ 10^12^ s^− 1^), $\:{K}_{B}$ the Boltzmann constant, T the temperature (298 K), and $\:A$ the Richardson constant (6 × 10^5^ A.m^− 2^.K^− 2^)^26^.

The dipole moment ($\:\mu\:$) and molecular polarizability ($\:\alpha\:$) of the studied systems were evaluated using Eqs. (7) and (8), respectively^27^.

7 $$\:\boldsymbol{\mu\:}=\sqrt{{\boldsymbol{\mu\:}}_{\boldsymbol{x}}^{2}+{\boldsymbol{\mu\:}}_{\boldsymbol{y}}^{2}+{\boldsymbol{\mu\:}}_{\boldsymbol{z}}^{2}}$$

8 $$\:\boldsymbol{\alpha\:}=\frac{1}{3}\left({\boldsymbol{\alpha\:}}_{\boldsymbol{x}\boldsymbol{x}}+{\boldsymbol{\alpha\:}}_{\boldsymbol{y}\boldsymbol{y}}+{\boldsymbol{\alpha\:}}_{\boldsymbol{z}\boldsymbol{z}}\right)$$

In Eq. (7), $\:\mu\:$ represents the total dipole moment of the system and is calculated from the vector sum of the dipole moment components along the three Cartesian axes ($\:{\mu\:}_{x}$, $\:{\mu\:}_{y}$, and $\:{\mu\:}_{z}$). These components describe the charge separation along the x, y, and z directions, respectively, and their combined magnitude determines the overall polarity of the molecule.

In Eq. (8), α represents the mean molecular polarizability, which describes the ability of the electron cloud of a molecule to be distorted under an external electric field. The parameters $\:{\alpha\:}_{xx}$, $\:{\alpha\:}_{yy}$, and $\:{\alpha\:}_{zz}$ correspond to the diagonal components of the polarizability tensor along the x, y, and z directions, respectively. The average polarizability is obtained from these tensor components.

Quantum Theory of Atoms in Molecules (QTAIM) analysis (Using the AIM2000 program (version 2.0)) was carried out to characterize the nature of the interactions between PCE and the fullerene-based sensors^28^. The topological properties of the electron density were analyzed at bond critical points (BCPs), including electron density ($\:\rho\:$), Laplacian of electron density ($\:{\nabla\:}^{2}\rho\:$), and kinetic and potential energy densities. These parameters provide quantitative information about the strength and character of intermolecular interactions. The QTAIM analysis was performed using the wavefunction obtained from the DFT calculations, allowing identification of interaction paths and detailed characterization of bonding between PCE and the sensor surface.

These calculations were performed to provide a detailed understanding of the molecular interactions between C_60_ (and its doped forms) and PCE, clarifying the nature, strength, and stability of these interactions at the atomic and electronic levels. Understanding these interactions computationally enables the identification of promising C_60_-based systems that could be translated into practical applications for PCE monitoring, detection, or remediation.

## Results and discussion

### Bond lengths/angles

When investigating the effects of doping, consideration needs to be given to bond lengths and bond angles since these structural parameters affect, in part, the electronic characteristics and reactivity of doped materials. When foreign atoms such as Al or Zn are added to a C_60_ structure through doping, distortion of the bond length and bond angle between the carbon atoms in the fullerene will occur. Such changes will result in changes to the electron density distribution in the carbon-carbon bonds, changes in charge-transfer behavior, and changes in the ability of the material to interact with target molecules^29^. Therefore, a computational study of the bond lengths and bond angles of each of the designed structures was conducted, and findings are presented in Table 1.

**Table 1:** Bond length/angle values (In Angstroms (Å)) ​​for C_60_, AlC_59_, and ZnC_59_ (In Degrees (D)).

Structure	Bond lengths (Å)			Bond angles (D)	
		Theoretical	Experimental		
C_60_	C_11_-C_1_	1.45	1.40–1.45 [30]	C_1_-C_11_-C_2_	120
	C_11_-C_2_	1.40		C_1_-C_11_-C_3_	108
	C_11_-C_3_	1.45		C_2_-C_11_-C_3_	119
	C_2_-C_6_	1.40		C_3_-C_4_-C_5_	108
	C_6_-C_7_	1.45		C_4_-C_5_-C_2_	108
	C_2_-C_5_	1.45		C_5_-C_2_-C_6_	120
	C_5_-C_4_	1.45		C_2_-C_6_-C_7_	120
	C_6_-C_8_	1.45		C_2_-C_6_-C_8_	120
	C_8_-C_10_	1.40		C_6_-C_8_-C_10_	120
	C_10_-C_9_	1.45		C_8_-C_10_-C_9_	120
	C_9_-C_5_	1.40		C_10_-C_9_-C_5_	120
AlC_59_	Al-C_1_	1.92	–	C_1_-Al-C_2_	92
	Al-C_2_	1.92	–	C_1_-Al-C_3_	104
	Al-C_3_	1.90	–	C_2_-Al-C_3_	104
	C_2_-C_6_	1.40	–	C_3_-C_4_-C_5_	115
	C_6_-C_7_	1.51	–	C_4_-C_5_-C_2_	115
	C_2_-C_5_	1.46	–	C_5_-C_2_-C_6_	118
	C_5_-C_4_	1.51	–	C_2_-C_6_-C_7_	125
	C_6_-C_8_	1.44	–	C_2_-C_6_-C_8_	119
	C_8_-C_10_	1.40	–	C_6_-C_8_-C_10_	120
	C_10_-C_9_	1.45	–	C_8_-C_10_-C_9_	119
	C_9_-C_5_	1.41	–	C_10_-C_9_-C_5_	120
ZnC_59_	Zn-C_1_	2.03	–	C_1_-Zn-C_2_	82
	Zn-C_2_	2.02	–	C_1_-Zn-C_3_	91
	Zn-C_3_	1.97	–	C_2_-Zn-C_4_	99
	C_2_-C_6_	1.39	–	C_3_-C_4_-C_5_	113
	C_6_-C_7_	1.51	–	C_4_-C_5_-C_2_	113
	C_2_-C_5_	1.44	–	C_5_-C_2_-C_6_	119
	C_5_-C_4_	1.49	–	C_2_-C_6_-C_7_	124
	C_6_-C_8_	1.45	–	C_2_-C_6_-C_8_	121
	C_8_-C_10_	1.39	–	C_6_-C_8_-C_10_	120
	C_10_-C_9_	1.45	–	C_8_-C_10_-C_9_	118
	C_9_-C_5_	1.41	–	C_10_-C_9_-C_5_	119

The optimized geometry of C_60_ obtained from the computational method shows very good agreement with the available experimental data. The calculated C-C bond lengths of 1.40 and 1.45 Å fall entirely within the experimentally reported range of 1.40–1.45 Å, demonstrating a clear overlap between theory and experiment. In particular, the shorter C_11_-C_2_ bond (1.40 Å) corresponds to the experimentally observed shorter bonds, while the C_11_-C_1_ and C_11_-C_3_ bonds (1.45 Å) match the longer bonds characteristic of the fullerene framework. This close correspondence indicates that the computational approach reliably reproduces the distinct bonding environments present in C_60_. Also, the bond angles in C_60_ between neighboring carbon atoms (C_1_-C_11_-C_2_, C_1_-C_11_-C_3_, and C_2_-C_11_-C_3_) range from 108° to 120°, indicating the characteristic sp^2^ hybridization of carbon atoms in a hexagonal arrangement typical of fullerene molecules.

In contrast, the AlC_59_ structure exhibits significant changes in bond lengths and angles due to Al incorporation. The Al-C bond lengths (1.90–1.92 Å) are notably longer than the C-C bonds in C_60_, indicating that the Al atom is larger and less covalently bonded to the carbon atoms. This elongation is expected because Al is a metal with a different atomic size and bonding characteristics compared to carbon. Additionally, the bond angles in AlC_59_ (92°-104°) deviate significantly from those in the C_60_ structure. These changes suggest that the Al doping introduces strain into the C_60_ framework, distorting the idealized bond angles in the original fullerene.

Similarly, the ZnC_59_ structure shows even larger alterations in bond lengths and angles. The Zn-C bond lengths range from 1.97 to 2.03 Å, which are substantially longer than the C-C bonds in C_60_ and also longer than the Al-C bonds in AlC_59_. This suggests that zinc, being larger than Al, has an even weaker bond with the carbon atoms in the fullerene. The bond angles in ZnC_59_ (82°-99°) are also quite different from those in C_60_, with some angles notably smaller than those in both C_60_ and AlC_59_. This indicates that zinc doping introduces even greater strain in the structure than Al doping.

In addition to the intrinsic structural features of pristine C_60_, the introduction of metal dopants significantly affects the geometry of the surrounding carbon framework. When one carbon atom in the fullerene cage is replaced by a metal atom, the neighboring C-C bonds experience local structural perturbations due to the larger atomic radius and different bonding characteristics of the dopant compared with carbon. As shown in Table 1, several C-C bonds located near the dopant undergo noticeable changes relative to pristine C_60_. For instance, the C_2_-C_6_ bond changes from 1.40 Å in C_60_ to about 1.40 Å in AlC_59_ and 1.39 Å in ZnC_59_, while the C_6_-C_7_ bond increases from 1.45 Å in C_60_ to approximately 1.51 Å in both doped structures. Similarly, the C2-C5 bond slightly increases from 1.45 Å in C_60_ to about 1.46 Å in AlC_59_ and decreases slightly to 1.44 Å in ZnC_59_. The C5-C4 bond also elongates from 1.45 Å in pristine C_60_ to around 1.51 Å in AlC_59_ and 1.49 Å in ZnC_59_. Additional nearby bonds such as C_6_-C_8_, C_8_-C_10_, C_10_-C_9_, and C_9_-C_5_ also exhibit minor variations compared with the pristine cage, reflecting the redistribution of structural strain within the carbon network after metal substitution.

The bond angles in the neighboring region are also influenced by the presence of the dopant atoms. While pristine C_60_ generally exhibits bond angles between approximately 108° and 120°, several C-C-C angles deviate from this range after doping. For example, the C_3_-C_4_-C_5_ angle increases from 108° in C_60_ to about 115° in AlC_59_ and 113° in ZnC_59_, and the C_4_-C_5_-C_2_ angle shows a similar increase. The C_5_-C_2_-C_6_ angle slightly decreases from 120° in C_60_ to about 118° in AlC_59_ and 119° in ZnC_59_. In addition, the angles C_2_-C_6_-C_7_ and C_2_-C_6_-C_8_ increase to approximately 125° and 119° in AlC_59_ and about 124° and 121° in ZnC_59_, respectively. Other angles in the neighboring region, including C_6_-C_8_-C_10_, C_8_-C_10_-C_9_, and C_10_-C_9_-C_5_, also display small deviations compared with pristine C_60_. These angular distortions confirm that the substitution of carbon with metal atoms breaks the high symmetry of the fullerene cage and introduces local strain into the carbon framework. Overall, these results clearly show that Al and Zn doping not only modifies the direct metal-carbon bonds but also induces measurable structural changes in the neighboring C-C bonds and C-C-C angles of the fullerene cage. Such local distortions redistribute electron density around the dopant site and create chemically active regions on the fullerene surface, which can enhance the interaction capability of the doped structures toward analyte molecules such as PCE.

To further clarify the electronic effect of metal doping, the atomic charge distribution of the carbon atoms in the fullerene cage was analyzed (Table 2). In pristine C_60_, the high symmetry of the structure results in nearly uniform charge distribution, with the carbon atoms exhibiting approximately neutral charges. However, substitution with metal dopants significantly perturbs this distribution. In the AlC_59_ system, the aluminum atom carries a strong positive charge (+ 0.410), indicating electron transfer from the metal center to the surrounding carbon framework. As a result, several neighboring carbon atoms become negatively charged (e.g., C_1_ = -0.139, C_2_ = -0.164, C_3_ = -0.164), while others exhibit small positive values due to charge redistribution within the cage. A similar but stronger effect is observed in ZnC_59_, where the zinc atom possesses an even larger positive charge (+ 0.545), accompanied by more pronounced negative charges on adjacent carbon atoms (e.g., C_1_ = -0.292, C_2_ = -0.260, C_3_ = -0.214). These results demonstrate that metal substitution induces significant charge polarization within the fullerene cage, creating electron-deficient centers at the dopant atoms and redistributing electron density over the carbon framework. Such charge redistribution is expected to enhance the interaction between the doped fullerene and electron-rich regions of the PCE molecule, thereby improving adsorption and sensing performance.

**Table 2:** Atomic charge distribution (e) for selected carbon atoms in pristine C_60_ and in Al- and Zn-doped fullerene structures (AlC_59_ and ZnC_59_), highlighting the charge redistribution induced by metal substitution.

C_60_										
C_1_	C_2_	C_3_	C_4_	C_5_	C_6_	C_7_	C_8_	C_9_	C_10_	C_11_
0.000	0.000	0.000	0.000	0.000	0.000	0.000	0.000	0.000	0.000	0.000

AlC_59_										
C_1_	C_2_	C_3_	C_4_	C_5_	C_6_	C_7_	C_8_	C_9_	C_10_	Al
− 0.139	− 0.164	− 0.164	0.015	0.015	0.014	0.008	0.011	0.002	− 0.011	0.410

ZnC_59_										
C_1_	C_2_	C_3_	C_4_	C_5_	C_6_	C_7_	C_8_	C_9_	C_10_	Zn
− 0.292	− 0.260	− 0.214	0.077	0.039	0.031	0.013	0.013	0.003	− 0.018	0.545

### Cohesive energy

**Fig. 2:**
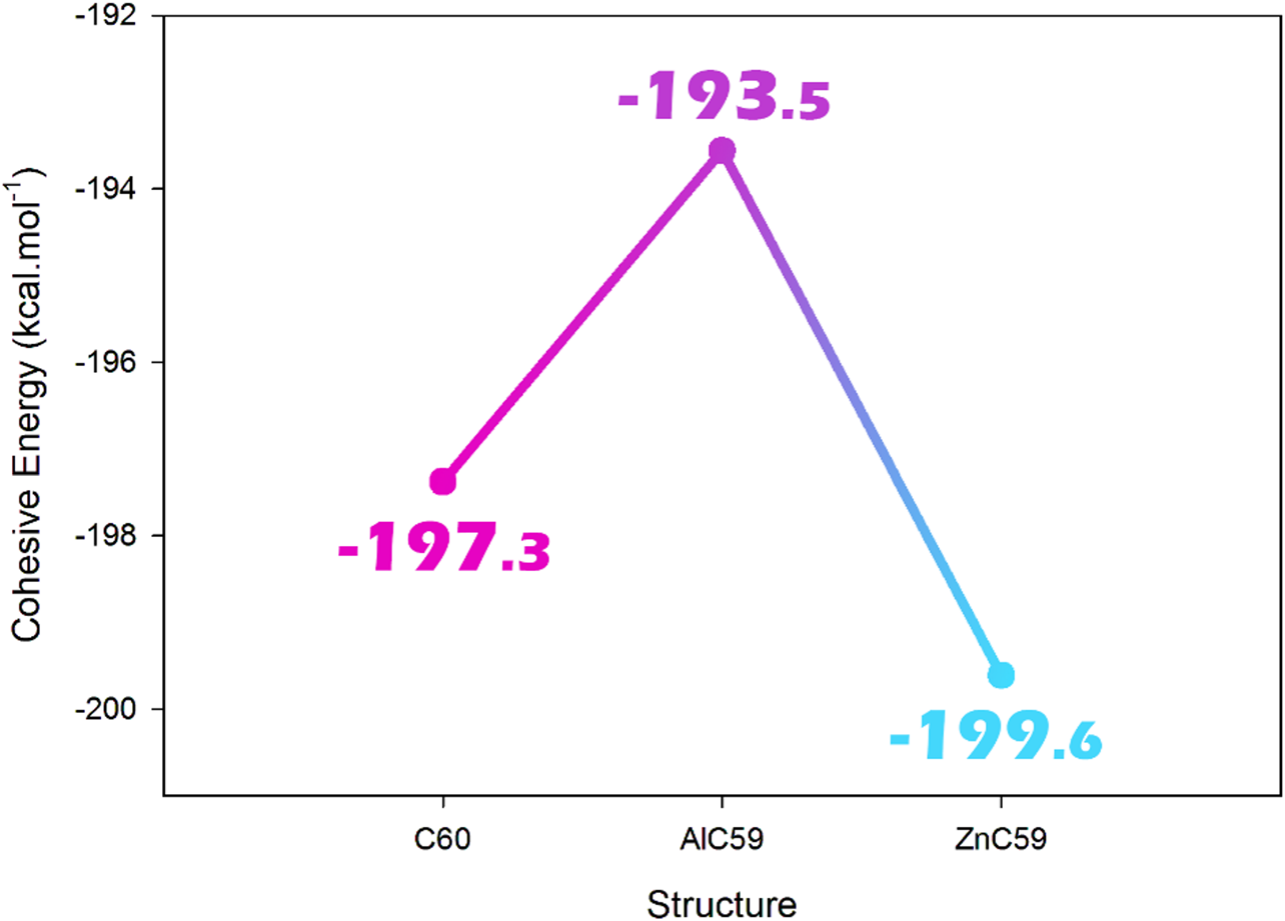
Cohesive energy changes after Al and Zn doping of pristine C_60_.

Cohesive energy refers to the energy required to break a solid into its constituent atoms or molecules and indicates the stability of a material and the degree to which its particles are strongly bound. A more negative cohesive energy corresponds to stronger overall atomic bonding and therefore greater intrinsic stability of the structure. Doping can significantly change the cohesive energy of a material. When a dopant, such as a metal atom, is introduced into a material such as C_60_, it can either enhance or weaken the cohesive energy of the material, depending on the nature of the doping^31^. Figure 2 presents the results of this investigation of this parameter.

The calculated cohesive energies show that pristine C_60_ possesses a value of − 197.3 kcal.mol^− 1^, reflecting the well-known stability of the fullerene cage. After metal substitution, different trends are observed for the two doped systems. The AlC_59_ structure exhibits a slightly less negative cohesive energy (-193.5 kcal.mol^− 1^), indicating a small reduction in structural stability compared with pristine C_60_. This behavior can be attributed to the larger Al-C bond lengths (1.90–1.92 Å) and the deviation of bond angles from the ideal fullerene geometry, which introduce local strain into the cage structure. In contrast, ZnC_59_ shows the most negative cohesive energy (-199.6 kcal.mol^− 1^), suggesting that zinc substitution slightly enhances the overall stability of the fullerene framework relative to both C_60_ and AlC_59_. Although the Zn-C bonds (1.97–2.03 Å) are longer than the original C-C bonds, the electronic interaction between Zn and the surrounding carbon atoms contributes to stronger overall binding within the system. Therefore, the cohesive energy results indicate that metal substitution affects the stability of the fullerene differently depending on the dopant, with Al slightly reducing and Zn slightly increasing the structural stability of the C_60_ cage.

### MEP contours

**Fig. 3:**
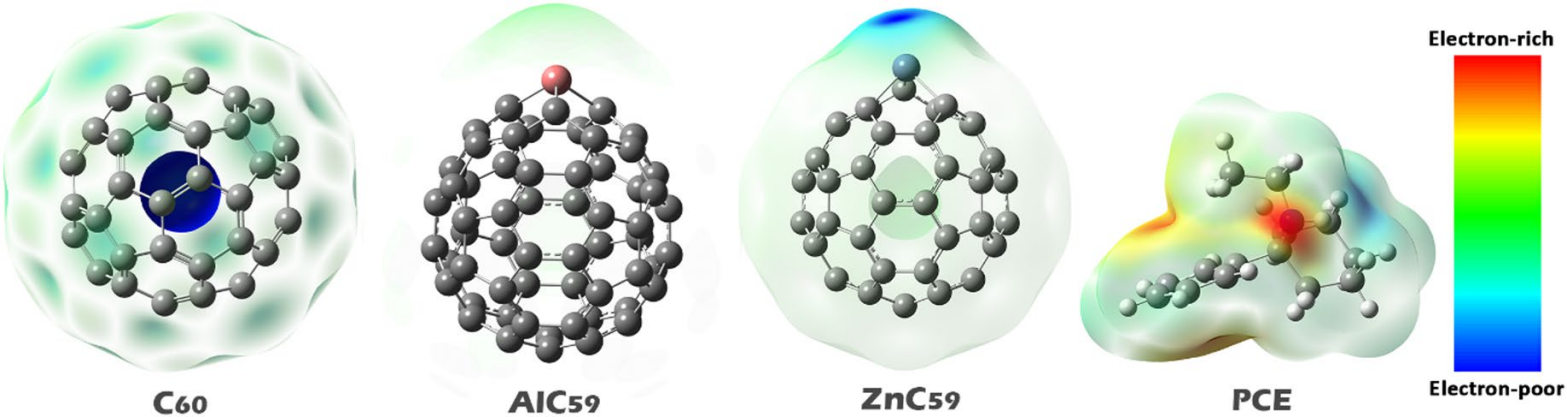
MEP contours for each of the structures studied in this work.

The study of Molecular Electrostatic Potential (MEP) contours for identifying potential sites for molecular interactions is a key consideration when developing compounds such as sensors and adsorbents. Additionally, MEP also provides insight into how molecules can easily interact with one another by showing areas of a molecule that are electron deficient (positive/electron-poor) and those that are electron rich (negative/electron-rich). The MEP contours also show the different electrostatic potentials throughout a given molecule. Areas shaded red on the MEP contours showed locations of strong electron density (negative potential) while the blue areas represent places of low electron density (positive potential) and the green/yellow contours represent areas of neutral/intermediate electrostatic potential^32^. The MEP contour for each molecule in isolation is presented in Fig. 3.

In the MEP contours of C_60_, the blue color, which represents regions of high positive potential (electron-deficient areas), is spread uniformly over the surface of the molecule. This suggests that C_60_ has a generally electron-poor surface, with no specific localized regions of strong positive potential. In contrast, when aluminum (Al) and zinc (Zn) are introduced into the C_60_ structure, as seen in AlC_59_ and ZnC_59_, the blue color becomes localized around the Al and Zn atoms. This indicates that the doping of C_60_ with these metals creates more concentrated electron-deficient regions specifically around the metal atoms. These localized positive regions can be highly reactive and more likely to interact with electron-rich species. In the case of PCE, the red color, which corresponds to regions of high negative potential (electron-rich areas), is concentrated on the nitrogen atom. This suggests that the nitrogen in PCE is the most electron-rich part of the molecule, making it a likely site for interactions with positively charged regions of other molecules. Although Al and Zn create localized positive regions, the magnitude of electrostatic potential alone does not fully determine interaction strength, and additional factors such as Lewis acidity and orbital interactions must also be considered when interpreting binding behavior.

**Fig. 4:**
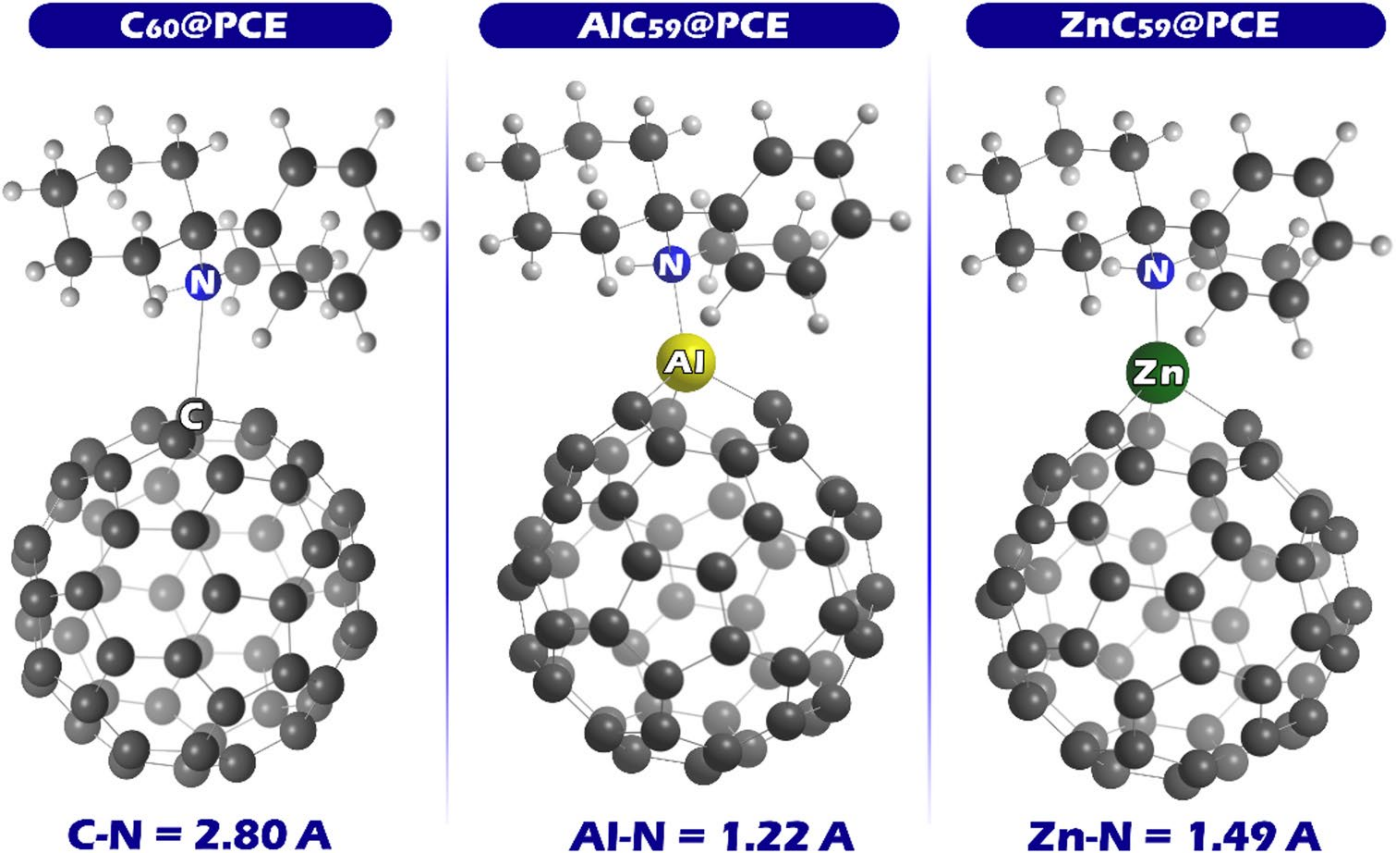
Optimized structure of each designed complex.

Based on these results, the interaction in the designed complex is more likely to occur through the metal atoms in AlC_59_ and ZnC_59_ (Al and Zn) and the nitrogen atom in PCE. The metal atoms in AlC_59_ and ZnC_59_ have a strong electron-deficient character, which can attract the electron-rich nitrogen atom of PCE. Therefore, these atoms (Al, Zn, and N) are expected to play a key role in the formation of the complex and in the interaction between the two molecules. According to these descriptions, each complex was designed. The optimal shape of each complex is shown in Fig. 4.

The bond length for each of the complexes is as follows: for C_60_@PCE, the C-N bond length is 2.80 Å, for AlC_59_@PCE, the Al-N bond length is 1.22 Å, and for ZnC_59_@PCE, the Zn-N bond length is 1.49 Å. Upon comparing these bond lengths, we observe significant differences in the strength of the interactions between PCE and the respective fullerene derivatives. In the C_60_@PCE complex, the bond length of 2.80 Å indicates a relatively weak interaction between the carbon and nitrogen atoms. This longer bond length suggests that the interaction between PCE and pristine C_60_ is weaker. In contrast, the AlC_59_@PCE complex shows a much shorter bond length of 1.22 Å between Al and N. This indicates a stronger and more stable interaction, as aluminum’s electron-deficient nature makes it more attractive to the electron-rich nitrogen in PCE. The shorter bond length implies that Al-doping significantly enhances the interaction between the fullerene derivative and PCE, forming a more robust complex. The ZnC_59_@PCE complex, with a bond length of 1.49 Å, lies between the interactions observed in the C_60_ and AlC_59_ complexes. While zinc also introduces an electron-deficient region, the bond length is not as short as that in AlC_59_, suggesting a moderately strong interaction. Although the MEP maps indicate that the Zn atom exhibits a slightly higher localized positive electrostatic potential than the Al atom, the interaction strength with PCE is not determined solely by the magnitude of the electrostatic potential. Factors such as Lewis acidity, orbital overlap, and structural geometry also play important roles. Aluminum, due to its stronger Lewis acidic character and more favorable orbital interaction with the nitrogen lone pair of PCE, forms a stronger interaction, which is reflected in the shorter Al–N bond distance. These results not only confirm that doping C_60_ with Al and Zn significantly improves its ability to interact with PCE, but also predict that the interaction is strongest in AlC_59_@PCE, followed by ZnC_59_@PCE, and the weakest in C_60_@PCE. However, these predictions require more detailed investigations, which will be discussed in detail in the following sections.

### Reactivity properties

Key reactivity properties such as energy gap, chemical hardness/softness; and chemical potential help to determine how a material will react with an analyte in the context of sensor design and analysis. Sensitivity is correlated to energy gap, with the smaller the energy gap, the more that sensor material will react with a particular analyte. Chemical hardness and chemical softness describe the resistance or ease with which a sensor material will exchange electrons with an analyte. Chemical potential describes the sensor’s tendency to donate or accept electrons. ECT parameters allow for prediction of charge transfer direction (i.e., whether the sensor will act as an electron donor or acceptor). In order to analyze sensor behavior upon exposure to analytes, the above properties must be examined both with and without an analyte for complete understanding of the sensor’s behaviors (see Table 3)^33,34^.

**Table 3:** The values ​​obtained for each of the reactivity parameters.

Structure	LUMO	HOMO	HLG	η	µ	S	$\:{\varDelta\:N}_{max}$	ECT
Sensor								
C_60_	− 3.58	− 5.25	1.67	0.83	− 4.41	0.59	5.28	**–**
AlC_59_	− 3.48	− 4.66	1.18	0.59	− 4.07	0.84	6.89	**–**
ZnC_59_	− 4.09	− 4.45	0.36	0.18	− 4.27	2.77	23.72	**–**
Complex								
C_60_@PCE	− 3.62	− 4.8	1.18	0.59	− 4.21	0.84	7.13	− 1.84
AlC_59_@PCE	− 3.04	− 4.12	1.08	0.54	− 3.58	0.92	6.62	− 0.26
ZnC_59_@PCE	− 3.92	− 4.24	0.32	0.16	− 4.08	3.12	25.5	− 1.77

**Fig. 5:**
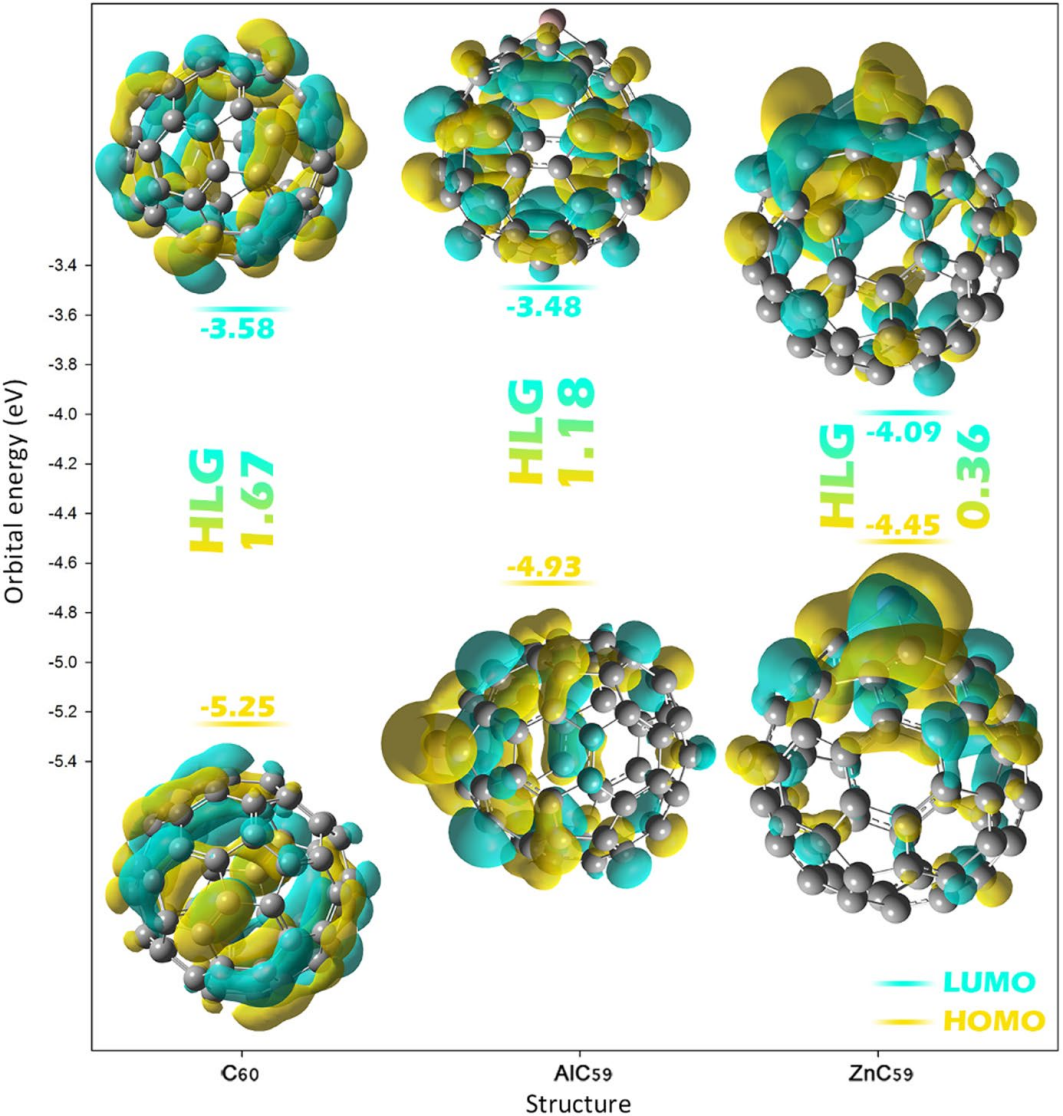
HOMO and LUMO energy levels and spatial distributions for C_60_, AlC_59_, and ZnC_59_, and the effect of metal doping on the HOMO-LUMO energy gap.

In the absence of PCE, C_60_ has an energy gap (HLG) of 1.67 eV, indicating moderate reactivity. The energy gap values calculated for C_60_ in this work agree well with experimental values reported in several studies, strengthening the validity of the computational approach used to investigate C_60_’s electronic properties and interactions. For instance, Rabenau et al. reported an energy gap of 1.85 eV for C_60_, determined from temperature-dependent microwave conductivity measurements^35^. This value is very close to our calculated energy gap, validating our computational method. Similarly, Kremer et al. conducted high-temperature conductivity studies on single-crystal C_60_ and found an energy gap of 1.86 eV, which also overlaps well with our result^36^. Additionally, Oshiyama et al. reported a slightly smaller energy gap of 1.50 eV for C_60_, based on their electronic-structure studies of fullerides^37^. While this value is somewhat lower than our calculated value, it is still within a reasonable range, reflecting the variability that can arise from different experimental methods and conditions (Fig. 5). Also, the chemical hardness of C_60_ (η = 0.83 eV) and chemical potential (µ = -4.41 eV) of C_60_ indicate that it is relatively stable and not highly reactive. The chemical softness (S = 0.59 eV^− 1^) is moderate, implying that C_60_ is neither too resistant nor too prone to changes in its electron density. The maximum charge transfer ($\:{\varDelta\:N}_{max}$ = 5.28) is also relatively low, indicating limited electron interaction capacity.

AlC_59_, on the other hand, has a smaller energy gap (HLG = 1.18 eV), suggesting it is more reactive than C_60_. Its lower chemical hardness (η = 0.59 eV) and slightly less negative chemical potential (µ = -4.07 eV) indicate it is more reactive and more likely to participate in charge transfer. The chemical softness (S = 0.84) is higher than that of C_60_, further confirming its increased reactivity, and its maximum charge-transfer ($\:{\varDelta\:N}_{max}$= 6.89) is higher, reflecting a greater capacity for interaction with other molecules. ZnC_59_ has the smallest energy gap (HLG = 0.36 eV), indicating it is highly reactive. It’s very low chemical hardness (η = 0.18 eV) and high chemical softness (S = 2.77 eV^− 1^) suggest it is highly susceptible to changes in electron density. Its maximum charge-transfer ($\:{\varDelta\:N}_{max}$= 23.72) is significantly higher than those of C_60_ and AlC_59_, confirming its enhanced reactivity and ability to engage in charge transfer.

When PCE is introduced into the system, the energy gaps of the complexes decrease slightly, indicating that PCE increases the sensors’ reactivity. C_60_@PCE has an energy gap (HLG = 1.18 eV), which is lower than that of the C_60_ sensor, suggesting increased reactivity upon complexation. The chemical hardness (η = 0.59 eV) and chemical potential (µ = -4.21 eV) remain similar to those in the sensor, but the chemical softness (S = 0.84 eV^− 1^) and maximum charge transfer ($\:{\varDelta\:N}_{max}$= 7.13) both increases, indicating a stronger interaction between C_60_ and PCE. The ECT value of -1.84 shows that charge is transferred from PCE to C_60_, as reflected by the negative value.

AlC_59_@PCE shows a further reduction in energy gap (HLG = 1.08 eV) and chemical hardness (η = 0.54 eV) compared to the AlC_59_ sensor, suggesting even greater reactivity upon interaction with PCE. Its chemical potential (µ = -3.58 eV) and chemical softness (S = 0.92 eV^− 1^) are also increased, and its maximum charge transfer ($\:{\varDelta\:N}_{max}$= 6.62) is slightly lower than in C_60_@PCE. The ECT value of -0.26 indicates that charge transfer from PCE to the sensor still occurs. ZnC_59_@PCE exhibits the smallest energy gap (HLG = 0.32 eV) and the lowest chemical hardness (η = 0.16 eV), suggesting it is the most reactive in the presence of PCE. The chemical softness (S = 3.12) is the highest among all complexes, reflecting its strong ability to engage in charge transfer. Its maximum charge transfer descriptor ($\:{\varDelta\:N}_{max}$= 25.5) is the highest among the investigated complexes. It should be noted that $\:{\varDelta\:N}_{max}$ is a dimensionless conceptual DFT parameter describing the theoretical maximum electron transfer capacity, rather than the actual number of electrons transferred between the fragments. The large value observed for ZnC_59_@PCE arises from the very small chemical hardness (η = 0.16 eV) and high electronic softness of the Zn-doped system, which mathematically increases the $\:{\varDelta\:N}_{max}$ descriptor. Therefore, this value should be interpreted as indicating enhanced charge-accepting ability and strong electronic responsiveness, rather than a literal transfer of tens of electrons. The ECT value of -1.77 suggests that charge transfer occurs from PCE to ZnC_59_, making it highly responsive to PCE. Finally, the data reveal that the introduction of Al and Zn doping significantly enhances the reactivity of C_60_ in the presence of PCE, with zinc doping providing the most favorable conditions for charge transfer.

**Fig. 6:**
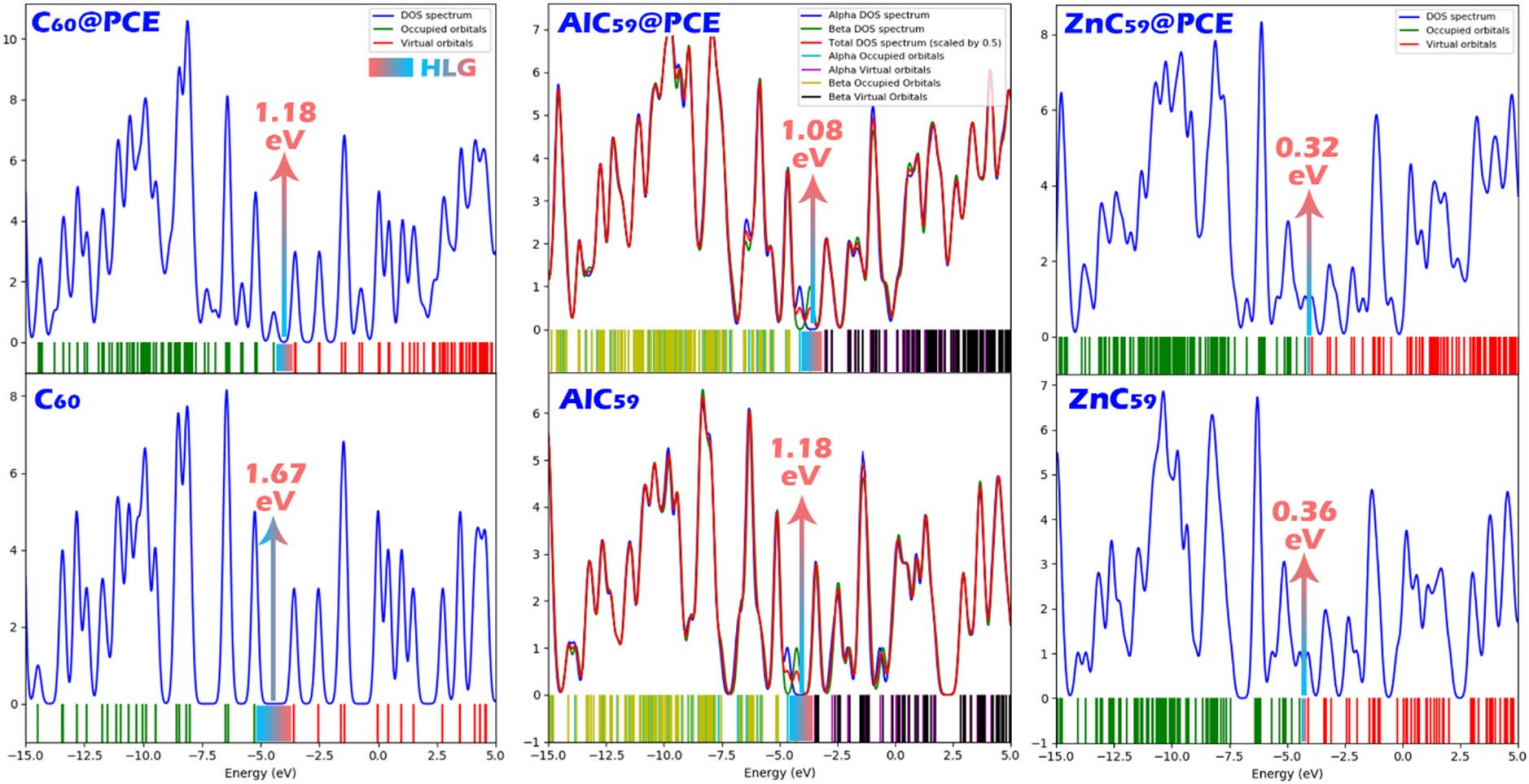
DOS plot for each of the studied sensors in the presence/absence of PCE.

Density of States (DOS) plots are an important visual tool for showing the distribution of electronic states in a molecule or material across different energy levels. These plots depict the number of available electronic states at each energy level, with the x-axis representing the energy and the y-axis representing the number of states (Fig. 6)^38^. Overall, the energy gaps indicated by the DOS plots closely match the values reported in Table 2, further validating the consistency and accuracy of both the computational methods used and the energy gap calculations for these structures and their complexes.

**Fig. 7:**
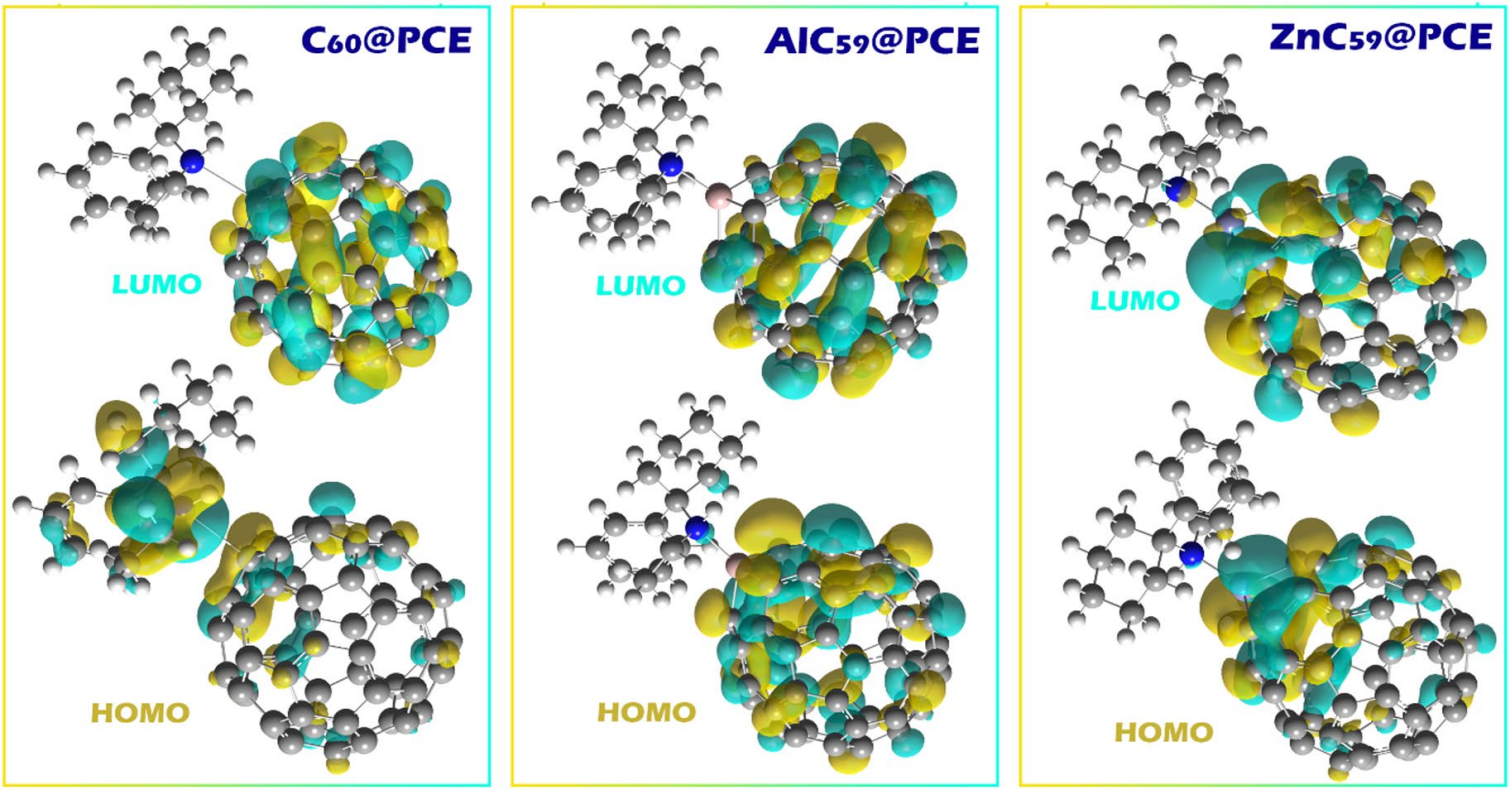
HOMO and LUMO orbital distributions for C_60_@PCE, AlC_59_@PCE, and ZnC_59_@PCE complexes, where the colored isosurfaces (yellow and cyan) represent opposite phases of the molecular orbitals.

The distribution of HOMO and LUMO orbitals in molecules is crucial because it dictates the material’s electronic properties, particularly its reactivity and ability to interact with other molecules (Fig. 7)^39^. The spatial distribution of frontier molecular orbitals provides qualitative insight into possible charge-transfer pathways between the analyte and the sensor surface. In the C_60_@PCE complex, the HOMO is mainly localized on the PCE molecule, while the LUMO is predominantly distributed over the C_60_ cage. This spatial separation of the frontier orbitals indicates a donor–acceptor configuration in which PCE acts as the electron donor and the fullerene surface behaves as the electron acceptor. Consequently, electrons located in the HOMO region of PCE can be transferred toward the LUMO region of C_60_ during interaction with the sensor surface. It should be noted that HOMO-LUMO visualization alone does not quantitatively measure charge transfer but instead indicates the most probable electron-transfer pathway. Therefore, this interpretation is supported by the calculated charge-transfer descriptors ($\:{\varDelta\:N}_{max}$ and ECT values in Table 2), which also indicate electron transfer from PCE toward the fullerene system. This interpretation is further supported by the DOS analysis and QTAIM/NCI results presented in other sections. The DOS plots show clear changes in the frontier electronic states after complex formation, indicating electronic interaction between PCE and the fullerene surface. Moreover, the QTAIM and NCI analyses reveal electron density redistribution and the formation of interaction pathways between the nitrogen atom of PCE and the active sites of the fullerene systems. Together, these results consistently support the occurrence of charge redistribution between PCE and the sensor structures. In AlC_59_@PCE and ZnC_59_@PCE, however, the HOMO and LUMO orbitals are both located on the sensor (AlC_59_ and ZnC_59_, respectively). This indicates that doping C_60_ with Al or Zn modifies its electronic properties, enabling it to both donate and accept electrons through its own orbitals. In these complexes, the sensor itself undergoes charge transfer in response to the analyte, rather than relying on interactions between HOMO and LUMO orbitals on different molecules. This change in orbital distribution likely enhances reactivity and strengthens interactions with PCE, making these doped structures more sensitive to electron exchange.

### Adsorption and sensor performance

One of the most significant factors affecting sensitivity, reversibility, and performance when designing a sensor includes adsorption energy, recovery time, and electrical conductivity. Adsorption energy indicates how strong an analyte will bond; therefore, an optimal equilibrium is necessary for effective detection and release. The recovery time will determine how long it takes for the sensor to reset itself before it can be reused and constantly monitored. Conductivity is very important because the measurable change in conductivity due to adsorbing analyte is the detection signal from the sensor^40,41^. All these parameters were reported in Table 4.

**Table 4:** Calculated values ​​of parameters $\:{\mathbf{E}}_{\mathbf{a}\mathbf{d}\mathbf{s}}$, $\:\boldsymbol{\tau\:}$, and $\:\boldsymbol{\sigma\:}$ (×10^9^) for each sensor in the presence/absence of PCE.

Structure	$\:{\mathrm{E}}_{\mathrm{a}\mathrm{d}\mathrm{s}}$ (kcal.mol^− 1^)	$\:\tau\:$ (s)	$\:\sigma\:$ (S/m)
C_60_	**–**	**–**	2.20
C_60_@PCE	− 11.15	1.54 × 10^− 4^	2.43
AlC_59_	**–**	**–**	2.43
AlC_59_@PCE	− 54.08	4.57 × 10^27^	2.48
ZnC_59_	**–**	**–**	2.87
ZnC_59_@PCE	− 32.78	1.09 × 10^12^	2.89

The data show that PCE adsorption on pristine C_60_ is relatively weak, with an adsorption energy of -11.15 kcal.mol^− 1^ for C_60_@PCE, and this is accompanied by an extremely short recovery time (1.54 × 10^− 4^ s). This combination is ideal for a sensor intended for repeated use, because the interaction is strong enough to produce a detectable response yet weak enough to allow rapid desorption and sensor regeneration. This interpretation is fully consistent with the complex bond-length result, where C_60_@PCE has the longest interaction distance (C-*N* = 2.80 Å), reflecting the weakest binding among the three complexes, which naturally supports fast recovery and reusability. At the same time, the electrical conductivity increases significantly after PCE adsorption, providing a clear conductivity-based sensing signal while maintaining reversibility, indicating that C_60_ is the best electrochemical sensor for repeated applications.

In contrast, AlC_59_@PCE exhibits a very strong interaction with PCE, as reflected by its much more negative adsorption energy (-54.08 kcal.mol^− 1^) and a very long recovery time (4.57 × 10^27^ s). Such behavior is unfavorable for a reusable sensor because the analyte would not desorb under practical conditions, but it is highly desirable for adsorption/removal purposes, since the pollutant would be strongly captured and effectively immobilized. This conclusion matches the bond-length evidence: AlC_59_@PCE shows the shortest interaction distance (Al-*N* = 1.22 Å), which is consistent with the strongest binding and, therefore, the slowest recovery. Although the conductivity of AlC_59_ changes slightly in the presence of PCE, the dominant feature here is strong adsorption and essentially irreversible adsorption, so AlC_59_ is better known as an adsorbent for PCE removal in environmental protection applications.

For completeness, ZnC_59_@PCE shows intermediate behavior, with adsorption energy (-32.78 kcal.mol^− 1^) and recovery time (1.09 × 10^12^ s) that are weaker and shorter than those of AlC_59_@PCE, but still far too strong/slow for practical repeated sensing. Also, the electrical conductivity of ZnC59 changes slightly in the presence of PCE, which does not seem suitable for use as an electrochemical sensor. This again aligns with the bond-length trend, since ZnC_59_@PCE has an intermediate interaction distance (Zn-*N* = 1.49 Å), giving binding stronger than C_60_@PCE but weaker than AlC_59_@PCE. Overall, the adsorption-energy/recovery-time ranking and the bond-length ranking are consistent (Al-N shortest/strongest > Zn-N intermediate > C-N longest/weakest), supporting the identification of C_60_ as the most reusable electrochemical sensor and AlC_59_ as the most effective adsorbent for PCE removal.

**Fig. 8:**
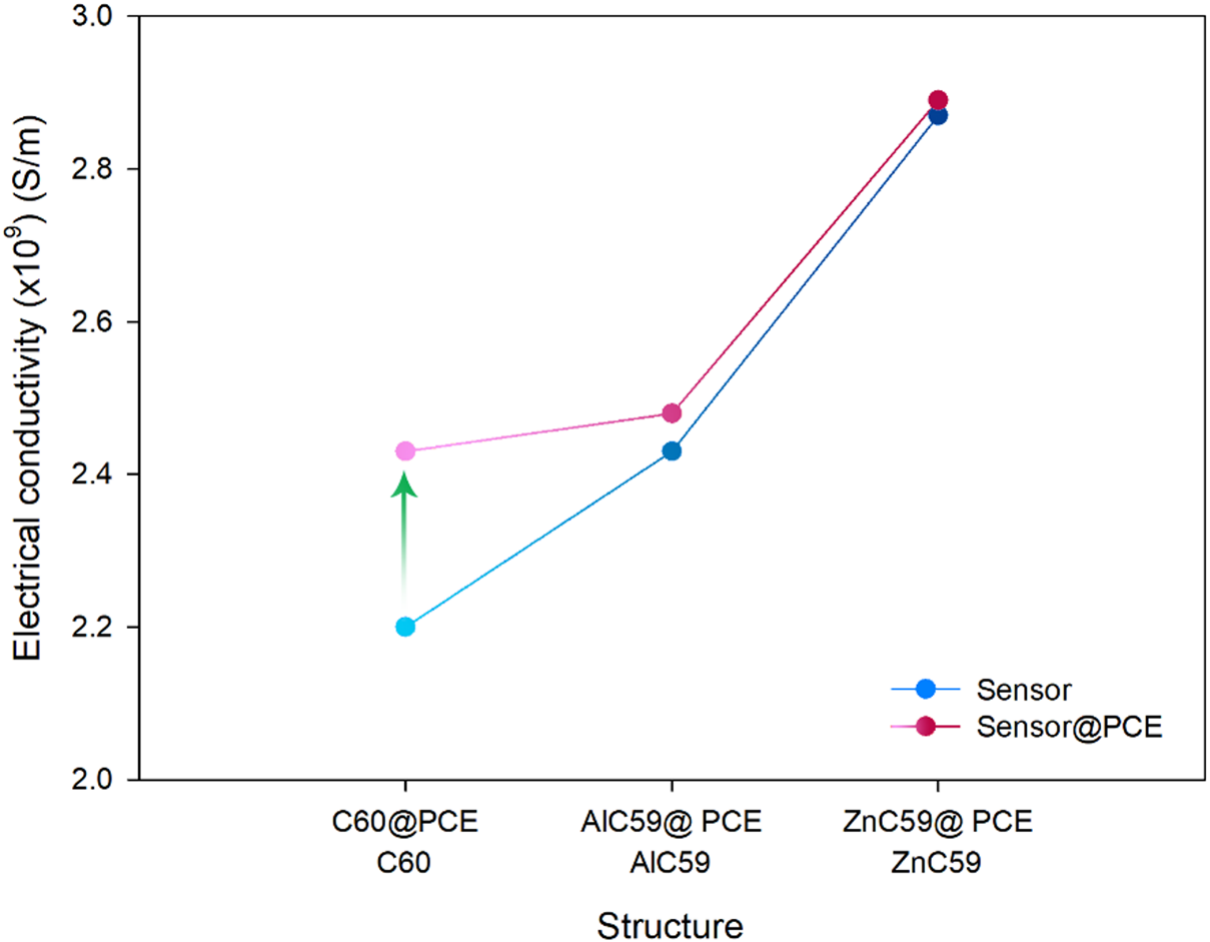
Qualitative representation of the trend of electrical conductivity for each of the designed sensors in the presence/absence of PCE rather than absolute quantitative values.

Figure 8 clearly shows the trend in electrical conductivity with/without PCE. Figure 8 is presented to emphasize qualitative trends rather than absolute quantitative values. This graph focuses on comparative measurement behavior among the designed systems rather than absolute numerical predictions. This figure visualizes the increase in electrical conductivity of C_60_ in the presence of PCE. It should be noted that the results reported in this study are based on computational predictions and should not be used as a substitute for absolute experimental values. Rather, they are intended for qualitative evaluation and comparison, and their validation requires future experimental studies. These results provide predictions of the behavior of each of the designed structures and pave the way for future experimental work.

**Fig. 9:**
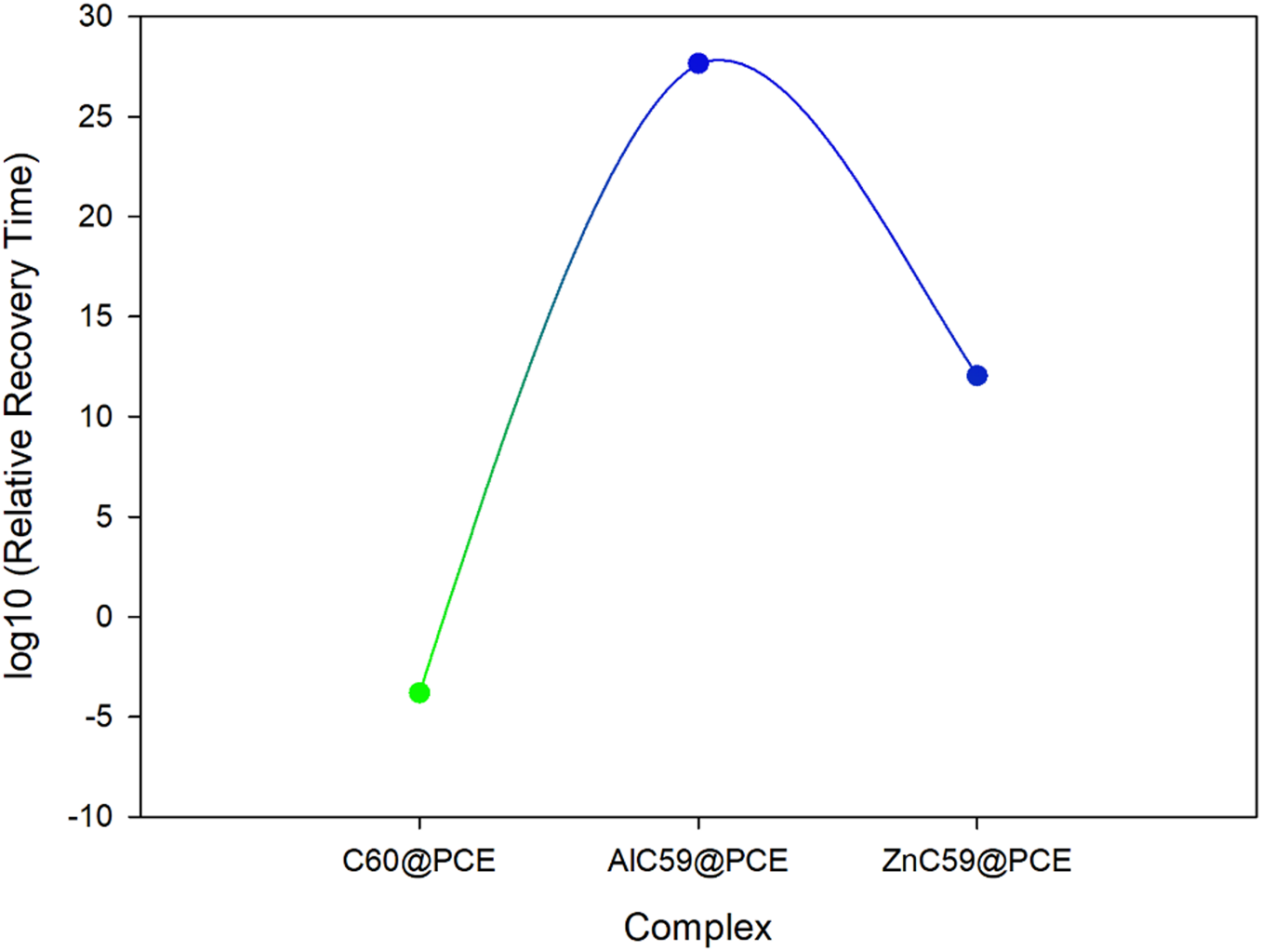
Logarithmic (log10) comparison of recovery time (τ) for C_60_-, AlC_59_-, and ZnC_59_-based systems upon PCE adsorption, illustrating relative desorption trends derived from computational analysis.

Figure 9 presents a logarithmic comparison of recovery time trends for the investigated systems following PCE adsorption. Among the complexes, AlC_59_@PCE exhibits a markedly longer recovery trend compared to ZnC_59_@PCE and C_60_@PCE, indicating a stronger interaction between the adsorbate and the doped surface. ZnC_59_@PCE shows an intermediate recovery behavior, while C_60_@PCE demonstrates the shortest recovery trend, suggesting comparatively faster desorption characteristics. It should be noted that these recovery times are obtained from computational calculations and are intended to represent qualitative, relative trends rather than exact experimental values.

Dipole moment and polarizability were investigated as important factors in evaluating sensor performance, as both strongly influence how a sensor responds to the presence of an analyte at the electronic level. The dipole moment reflects the degree of charge separation within a molecule or complex, and changes in dipole moment upon analyte adsorption indicate charge redistribution between the sensor and the analyte. Such charge redistribution directly affects the local electric field at the sensor surface, which is crucial for generating measurable electrochemical signals. A higher or significantly altered dipole moment after adsorption generally leads to stronger interactions with the external electric field and electrodes, enhancing signal intensity and sensitivity. Polarizability, on the other hand, describes how easily the electron cloud of a molecule can be distorted in response to an external electric field or to interaction with an analyte. A higher polarizability means the sensor’s electron density responds more readily to adsorption events, leading to pronounced changes in electronic properties such as conductivity and current flow. When an analyte binds to a highly polarizable sensor, even small interactions can induce significant electronic perturbations, thereby amplifying electrochemical responses^42^. For this purpose, each parameter was subjected to a computational study (Table 5).

**Table 5:** Values of Dipole moments ​​and polarizability for each designed structure.

Structure	Dipole moments (Debye)	Polarizability (a.u.)
C_60_	0.00	163.96
C_60_@PCE	2.26	685.88
AlC_59_	11.43	289.93
AlC_59_@ PCE	13.34	693.67
ZnC_59_	8.20	303.11
ZnC_59_@ PCE	8.74	705.40

The data in Table 4 show apparent differences in how dipole moment and polarizability change upon PCE adsorption on each structure. Pristine C_60_ has a dipole moment of 0.00 D, consistent with its high symmetry. Still, after complexation, the dipole moment rises sharply to 2.26 D in C_60_@PCE, indicating notable charge redistribution and polarization induced by the analyte. At the same time, the polarizability increases dramatically from 163.96 a.u. to 685.88 a.u., indicating that the electronic cloud of the C_60_-based system is more readily distorted in the presence of PCE, which is favorable for generating stronger electrical responses in electrochemical detection.

For the doped systems, AlC_59_ already exhibits a significant intrinsic dipole moment (11.43 D) due to symmetry breaking and charge localization around the dopant, and this value increases to 13.34 D upon adsorption (AlC_59_@PCE), confirming further charge rearrangement after interaction with PCE. Its polarizability also increases strongly from 289.93 a.u. to 693.67 a.u., indicating substantial electronic perturbation upon complex formation. Similarly, ZnC_59_ shows a relatively high dipole moment even before adsorption (8.20 D), which changes only slightly to 8.74 D in ZnC_59_@PCE, suggesting a smaller dipole-response to PCE compared with the Al-doped case. At the same time, its polarizability still increases markedly from 303.11 a.u. to 705.40 a.u., reflecting strong overall electronic responsiveness after adsorption.

**Fig. 10:**
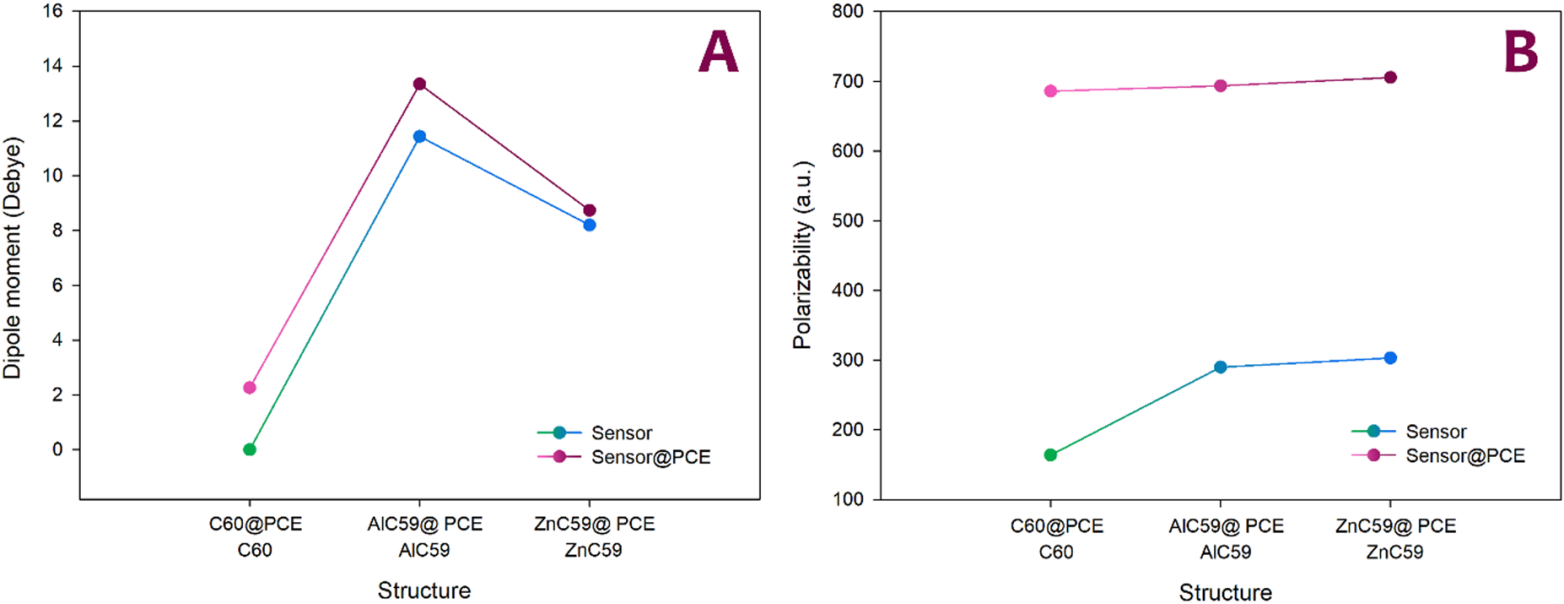
It visualizes (**a**) the dipole moment changes and (**b**) the trend of polarizability changes for each of the studied structures.

Comparing the complexes, all three show high polarizabilities in a narrow range (about 686–705 a.u.), meaning PCE adsorption leads to highly deformable electron density in every case; however, the most striking relative change is observed for C_60_, where polarizability increases by more than fourfold and the dipole moment shifts from zero to a clearly nonzero value. Therefore, C_60_ shows particularly significant electronic changes in the presence of PCE, consistent with the earlier observation that its electrical conductivity increases upon PCE adsorption, since stronger polarization and charge redistribution typically yield a clearer conductivity-based electrochemical signal. To better understand the process of changes in dipole moment and polarizability in each of the studied structures, see Fig. 10.

### UV–Vis spectrum

Sensor design relies heavily on the UV-Vis spectrum for the analysis of variations in electronic structure of materials due to interaction(s) with analyte(s) To indicate high interaction levels and a high probability of charge transfer, shifts in the maximum absorption wavelength (λmax) indicate strong interactions and result in the use of optical signals for sensor detection. Excitation energy (Eex) is determined by the required energy for electronic transitions^43^. To determine sensitivity and selectivity of a given sensor, λmax and Eex values must be compared for the same sensor when there is no analyte present and then for the same sensor with the presence of an analyte, as shown in Table 6.

**Table 6:** Theoretical and experimental (Taken from other literature) maximum absorption wavelengths (λmax) and corresponding excitation energies (Eex) of C_60_, metal-doped fullerenes, and their PCE complexes, highlighting the close agreement between theoretical and experimental data for C_60_ and the pronounced red shifts induced by PCE interaction.

Structure	λ_max_ (nm)	Eex (eV)
C_60_	360 (Theoretical)	2.78
	345 (Experimental)^44^	
C_60_@PCE	530	2.33
AlC_59_	521	2.37
AlC_59_@PCE	765	1.61
ZnC_59_	427	2.89
ZnC_59_@PCE	764	1.62

For C_60_, the theoretical absorption maximum at 360 nm (excitation energy 2.78 eV) is very close to the experimental value ​​at 345 nm. This small deviation is well within the typical accuracy of theoretical approaches and indicates that the computational model reliably reproduces the experimental optical response of C_60_.

Upon interaction with PCE, a pronounced red shift is observed. The λmax of C_60_@PCE moves to 530 nm, accompanied by a reduction in excitation energy to 2.33 eV, indicating enhanced charge-transfer interactions and improved light-harvesting in the visible region. A similar trend is observed for the metal-substituted fullerenes. AlC_59_ shows an absorption at 521 nm (2.37 eV), which shifts dramatically to 765 nm (1.61 eV) after complexation with PCE. ZnC_59_ behaves somewhat differently in its isolated form, absorbing at 427 nm with a higher excitation energy of 2.89 eV, but upon interaction with PCE it also exhibits a strong red shift to 764 nm and a markedly reduced excitation energy of 1.62 eV.

**Fig. 11:**
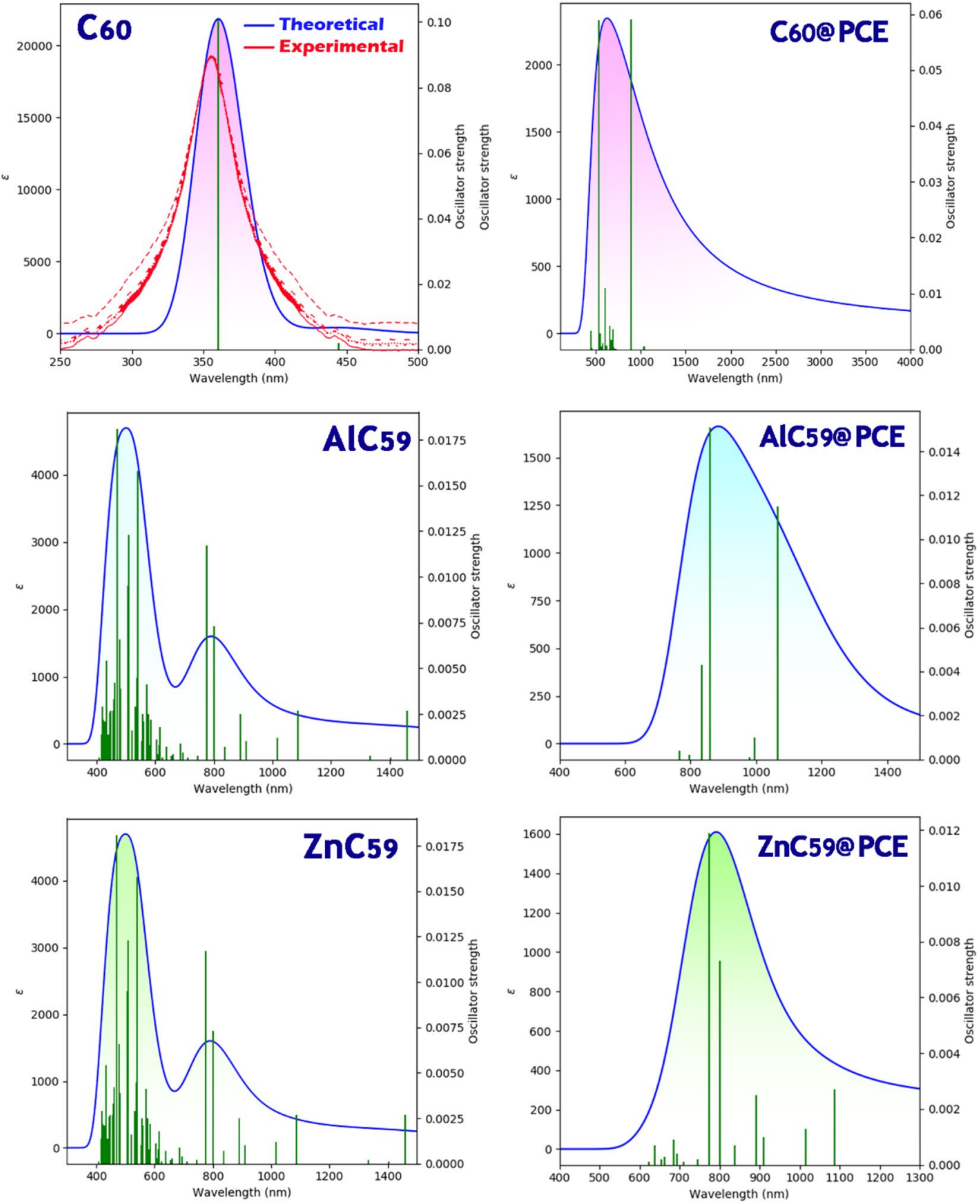
Qualitative display of the trend of λmax changes instead of numerical values ​​for each of the designed sensors in the presence/absence of PCE.

Comparing all PCE-based complexes, AlC_59_@PCE and ZnC_59_@PCE display the most significant bathochromic shifts and the lowest excitation energies, extending absorption deep into the near-infrared region. Between them, AlC_59_@PCE shows the lowest excitation energy (1.61 eV) and a λmax comparable to ZnC_59_@PCE, suggesting slightly stronger electronic coupling with PCE. On this basis, AlC_59_@PCE can be identified as the best colorimetric sensor for PCE, as it offers the most pronounced optical response and the greatest sensitivity through a large, easily detectable shift in absorption wavelength. Figure 11 shows a qualitative comparison for each of the designed sensors in the presence and absence of PCE.

### NCI/RDG analysis

Designing sensor-analyte complexes necessitates the inclusion of NCI/RDG (Non-Covalent Interaction/Reduced Density Gradient) analysis as it visualizes the weak interactions that dictate how analytes will be bound to a sensor’s surface, thus impacting how an analyte will be detected. NCI/RDG provides a means for identifying and characterizing the non-covalent forces (e.g., van der Waals, hydrogen bonds, electrostatic-association) responsible for binding an analyte to the surface of a sensor, as well as providing information regarding the location, type, and associated strength of these interactions so that the strength of the interaction between the sensor and the analyte can be verified to be sufficient for detection while also establishing that the interaction will allow for sensor reuse^45^.

**Fig. 12:**
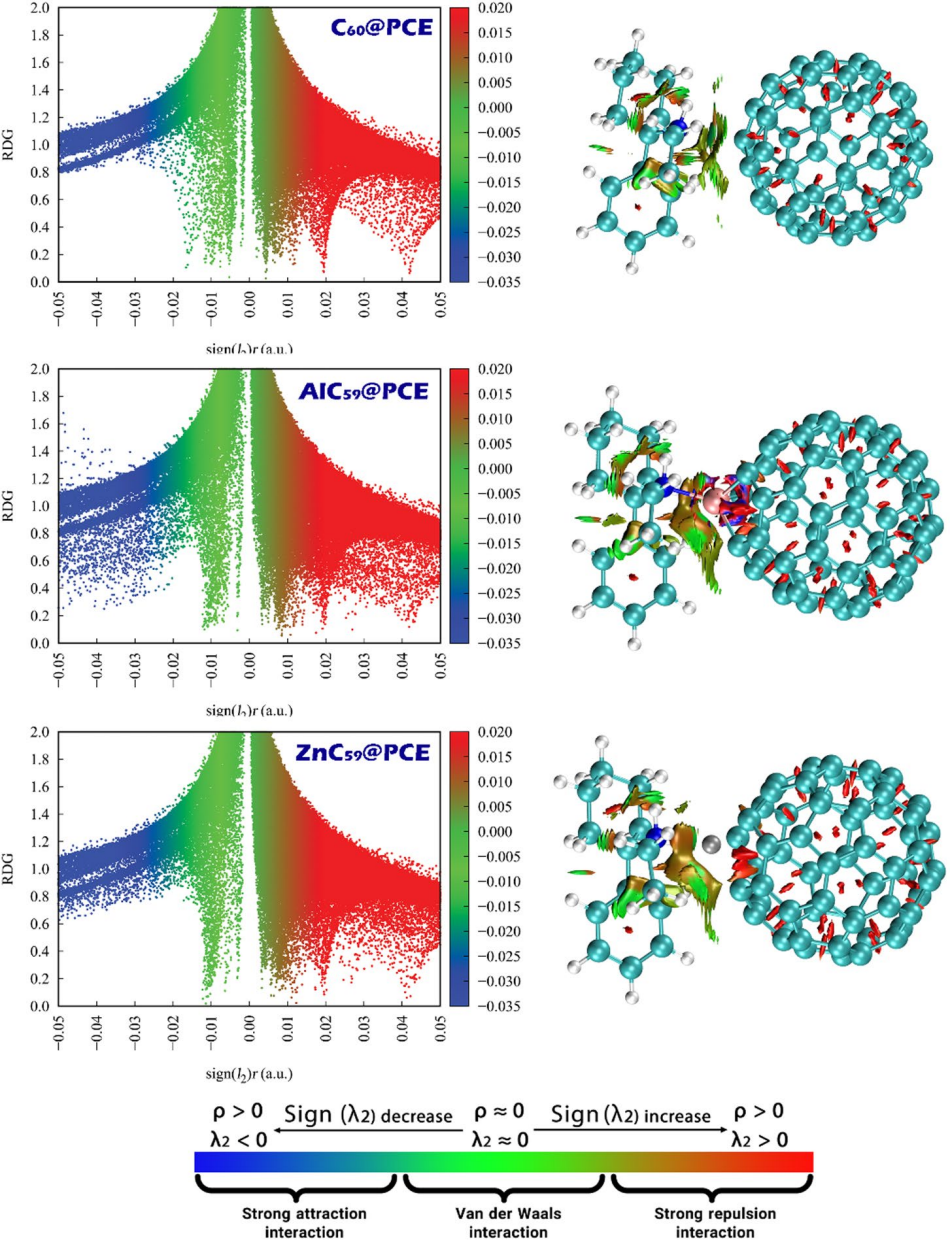
NCI/RDG analysis of C_60_@PCE, AlC_59_@PCE, and ZnC_59_@PCE complexes, illustrating the nature and strength of noncovalent interactions, where blue, green, and red regions correspond to attractive, van der Waals, and repulsive interactions, respectively.

The NCI/RDG plots in Fig. 12 provide a clear visual representation of the nature and strength of interactions between PCE and the three sensor systems, and they are fully consistent with previously reported adsorption energy data. In these plots, regions with blue coloring (λ_2_ < 0) correspond to strong attractive interactions, green regions (λ_2_ ≈ 0) indicate weak van der Waals interactions, and red regions (λ_2_ > 0) represent steric repulsion.

For C_60_@PCE, the RDG scatter plot and isosurface mainly show green regions between PCE and the C_60_ surface, with very limited blue areas. This indicates that the interaction is dominated by weak van der Waals forces rather than strong attractive bonding. This observation matches well with the relatively small adsorption energy reported for C_60_@PCE (-11.15 kcal.mol^− 1^), confirming a weak and reversible interaction, which is desirable for repeated sensor applications. In the case of AlC_59_@PCE, the NCI/RDG analysis reveals pronounced blue regions localized around the Al-N interaction zone, indicating strong attractive non-covalent interactions. These strong interaction features are much more intense and concentrated than those seen in C_60_@PCE. This directly correlates with the highly negative adsorption energy of -54.08 kcal.mol^− 1^, confirming that PCE binds very strongly to the Al-doped fullerene. Such strong attraction supports the interpretation of AlC_59_ as an effective adsorbent rather than a reusable sensor. For ZnC_59_@PCE, the NCI/RDG plots show a combination of blue and green regions, suggesting moderately strong attractive interactions accompanied by van der Waals contributions. The interaction strength is clearly stronger than in C_60_@PCE but weaker than in AlC_59_@PCE. This trend aligns well with the intermediate adsorption energy of -32.78 kcal.mol^− 1^, placing ZnC_59_@PCE between the other two systems in terms of binding strength.

The NCI/RDG analysis strongly supports the adsorption energy results, following the same interaction strength order: AlC_59_@PCE > ZnC_59_@PCE > C_60_@PCE. The dominance of van der Waals interactions in C_60_@PCE explains its weak adsorption energy and fast recovery, while the strong attractive interactions in AlC_59_@PCE justify its large adsorption energy and suitability for PCE removal applications.

### LOL and ELF map

Examining LOL (Localized Orbital Locator) and ELF (Electron Localization Function) contours is important because they provide real-space insight into how electrons are distributed and localized within molecular complexes, which helps clarify the nature of interactions between the adsorbate and the substrate. These analyses go beyond energy values by visually revealing whether bonding arises from covalent sharing, polarization, or weak noncovalent interactions. ELF primarily highlights regions of electron pair localization, making it especially useful for identifying covalent bonds, lone pairs, and bond strength, while LOL emphasizes the degree of orbital localization and is more sensitive to changes in delocalization and weak interactions. As a result, ELF is more effective for distinguishing strong, localized bonding, whereas LOL better captures subtle charge redistribution and interaction pathways, and together they provide a complementary and comprehensive picture of interaction mechanisms in complexes. For this purpose, the LOL and ELF contours for each of the designed complexes were computationally studied^46,47^.

**Fig. 13:**
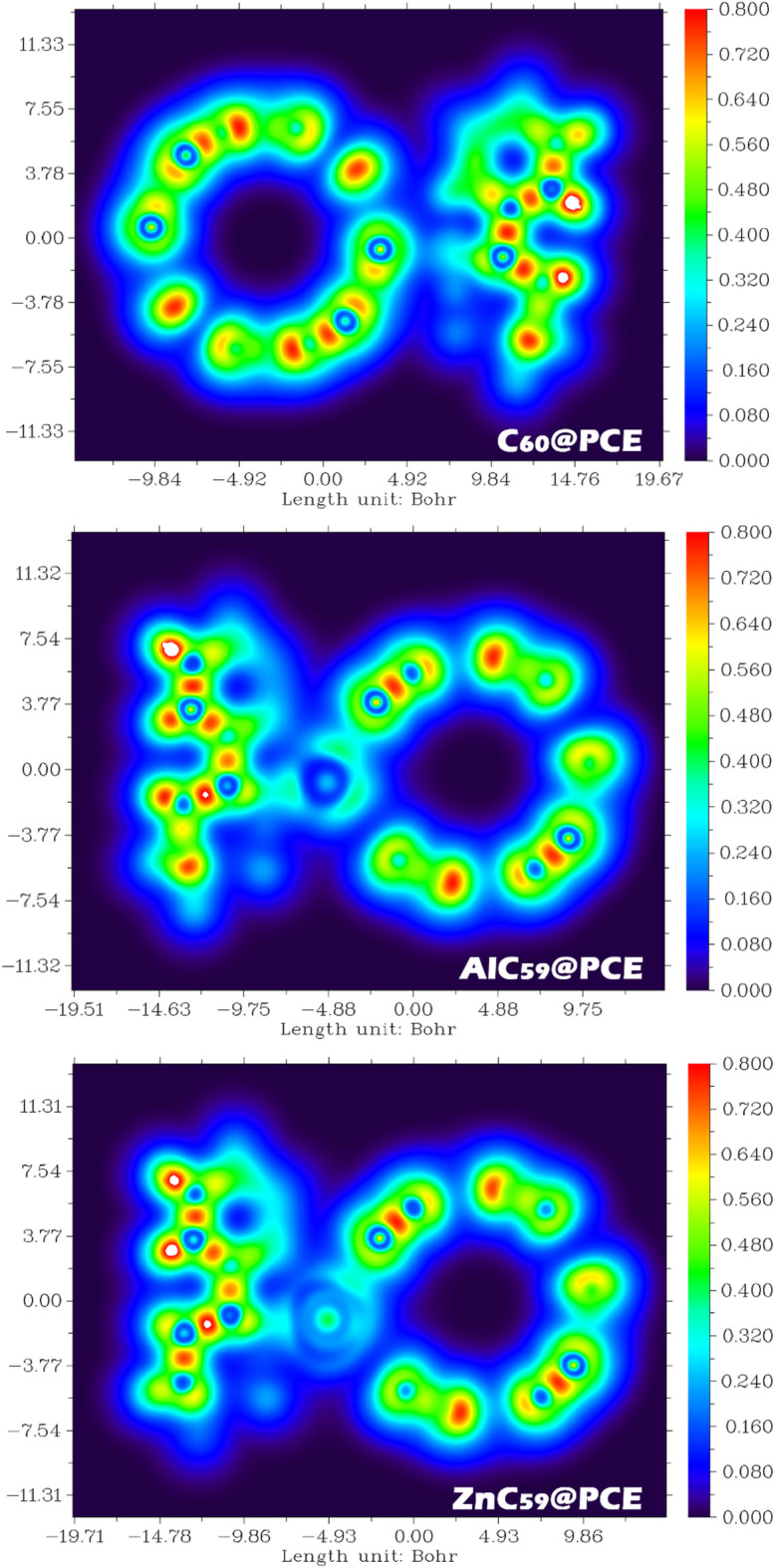
Localized orbital locator (LOL) contour maps of the PCE-adsorbed fullerene systems: C_60_@PCE, AlC_59_@PCE, and ZnC_59_@PCE.

High LOL values (typically shown by red–yellow regions) indicate strongly localized electrons associated with covalent bonds, lone pairs, or localized interaction regions, whereas low LOL values (blue regions) correspond to delocalized electrons or weakly interacting zones. The appearance, continuity, and intensity of LOL basins between fragments are therefore indicative of the interaction strength and character: continuous or intensified basins suggest stronger orbital overlap and charge localization, while separated or diffuse basins point to weak, noncovalent interactions dominated by dispersion or electrostatics (Fig. 13).

For C_60_@PCE, the LOL map shows that high localization regions are largely confined within the individual C_60_ cage and the PCE molecule, with no pronounced continuous high-LOL basin bridging the two fragments. The interaction region is characterized by relatively diffuse, low-to-moderate LOL values, indicating weak orbital overlap and limited charge localization at the interface. This pattern is consistent with a weakly interacting, physisorption-dominated complex. In AlC_59_@PCE, the LOL contours exhibit a marked enhancement of electron localization in the interfacial region near the Al site. Localized basins with higher LOL values extend toward the PCE moiety, suggesting significant orbital interaction and polarization induced by the aluminum dopant. The partial continuity of localized regions between AlC_59_ and PCE reflects stronger interaction and localized charge accumulation, consistent with chemisorption-like behavior. For ZnC_59_@PCE, the LOL distribution also shows increased localization at the contact region compared to pristine C_60_@PCE, but the extent and intensity of the interfacial basins are less pronounced than in the Al-doped system. The localized regions near the Zn site indicate dopant-assisted interaction and charge redistribution, yet the absence of a strongly continuous high-LOL basin suggests an interaction strength intermediate between C_60_@PCE and AlC_59_@PCE.

High ELF values (typically shown as red or yellow regions) correspond to strongly localized electron pairs associated with covalent bonds, lone pairs, or core electrons, whereas low ELF values (green to blue regions) indicate delocalized electrons characteristic of metallic bonding, π-delocalization, or weak noncovalent interactions. The shape, intensity, and continuity of ELF basins between interacting fragments provide direct insight into whether an interaction is covalent, polarized, or predominantly noncovalent.

For C_60_@PCE, the ELF contours show that regions of high electron localization are mainly confined within the individual C-C bonds of the fullerene cage and within the PCE molecule. The interfacial region between C_60_ and PCE lacks a distinct shared high-ELF basin, indicating the absence of significant electron-pair sharing. This suggests that the interaction is dominated by weak van der Waals forces and electrostatic contributions, consistent with physisorption. In AlC_59_@PCE, the ELF map reveals pronounced localization around the Al atom and noticeable polarization of electron density toward the PCE moiety. The appearance of enhanced ELF values in the vicinity of the Al-PCE contact region indicates stronger interaction accompanied by partial electron sharing and charge redistribution. This behavior reflects a chemisorption-like interaction in which the Al dopant acts as an active site, strengthening binding through localized electron pairing and polarization effects. For ZnC_59_@PCE, the ELF contours also display increased localization near the Zn center compared with pristine C_60_@PCE, confirming dopant-induced interaction enhancement. However, the localized basins are more confined around the Zn atom and adjacent atoms, with limited extension into the PCE fragment. This indicates a moderately strong, polarized interaction that is weaker than in the Al-doped system but stronger than in the undoped fullerene complex.

**Fig. 14:**
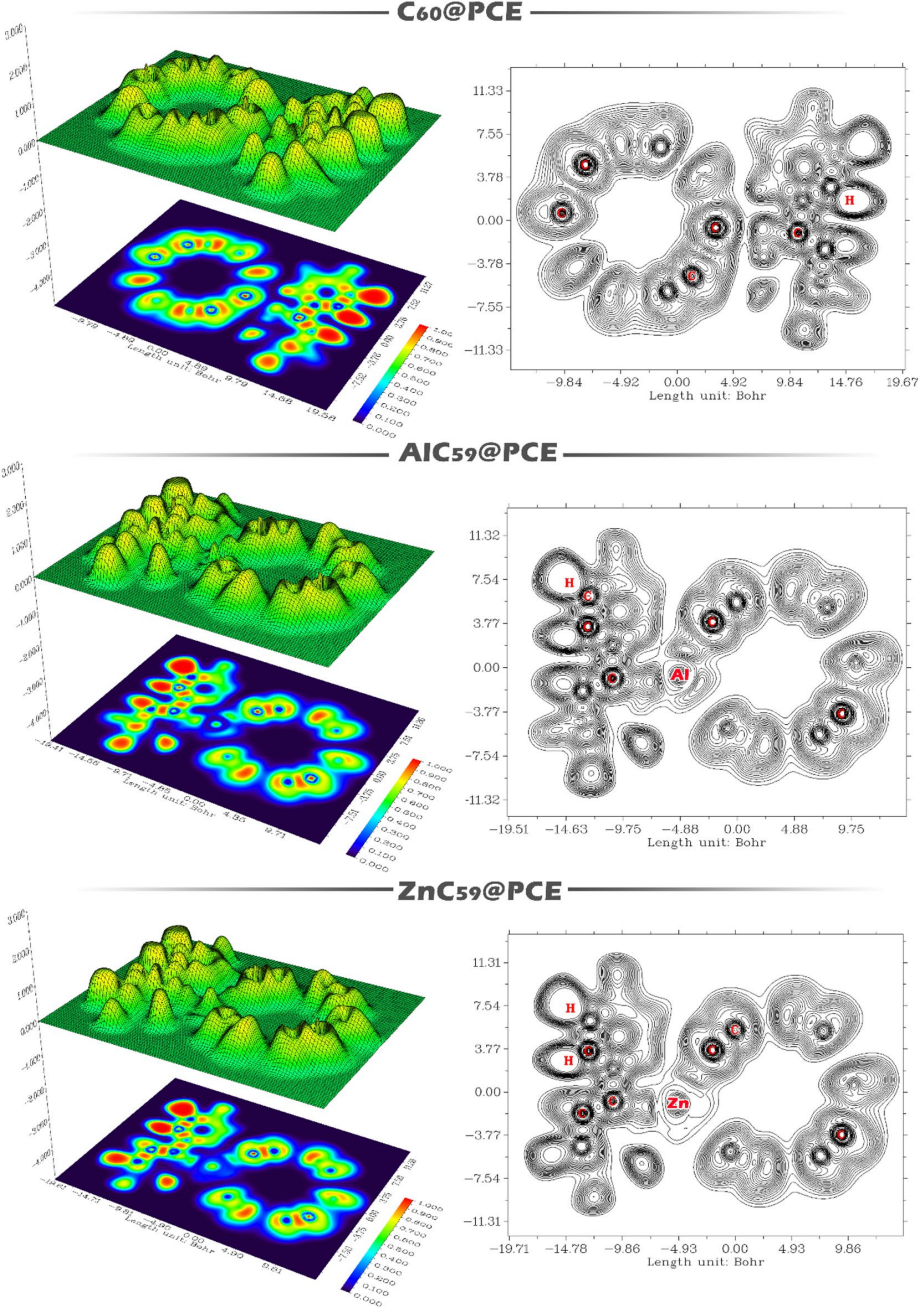
Three-dimensional surfaces (Left) and two-dimensional (Right) contour plots of the electron localization function (ELF) for C_60_@PCE, AlC_59_@PCE, and ZnC_59_@PCE.

Overall, the LOL and ELF analysis clearly differentiates the complexes: weak and nonlocalized interaction in C_60_@PCE, strong and highly localized interaction in AlC_59_@PCE, and moderate, dopant-enhanced localization in ZnC_59_@PCE. These trends corroborate the adsorption energy and recovery time results, confirming that metal doping intensifies orbital localization and interaction strength with PCE, with aluminum having the most pronounced effect (Fig. 14).

### QTAIM analysis

**Fig. 15:**
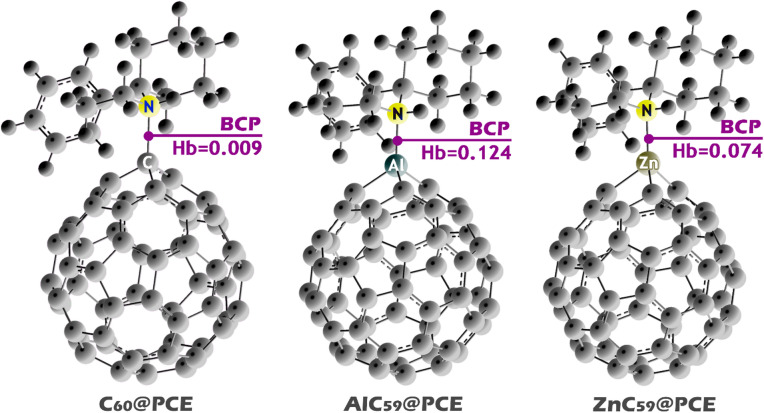
Bond critical point (BCP) visualization for C_60_@PCE, AlC_59_@PCE, and ZnC_59_@PCE complexes, showing the interaction paths between the PCE nitrogen atom and the sensor sites along with the corresponding Hb values.

The use of QTAIM (Quantum Theory of Atoms in Molecules) is important for studying interactions in molecular complexes because it provides a rigorous, electron-density-based framework for characterizing bonding and noncovalent interactions through bond-critical-point (BCP) properties (Fig. 15). By analyzing the electron density ($\:\rho\:\left(r\right)$) and its Laplacian ($\:{\nabla\:}^{2}\rho\:\left(r\right)$) at BCPs, QTAIM allows direct identification of interaction paths between atoms and clarifies whether an interaction is covalent, partially covalent, or purely noncovalent in nature. BCP parameters, such as electron density, Laplacian, and energy density, provide quantitative insight into the strength and stability of interactions, especially for weak interactions that are not easily described by geometric criteria alone^48^.

**Table 7:** QTAIM bond critical point (BCP) parameters for C_60_@PCE, AlC_59_@PCE, and ZnC_59_@PCE complexes, including electron density ($\:\boldsymbol{\rho\:}\left(\boldsymbol{r}\right)$),Laplacian ($\:{\nabla\:}^{2}\boldsymbol{\rho\:}\left(\boldsymbol{r}\right)$), and energy densities ($\:\boldsymbol{G}\left(\boldsymbol{r}\right)\:$and$\:\:\boldsymbol{V}\left(\boldsymbol{r}\right)$), highlighting the relative strength and nature of Sensor@PCE interactions.

Structure	$\:\rho\:\left(r\right)$	$\:{\nabla\:}^{2}\rho\:\left(r\right)$	$\:G\left(r\right)$	$\:V\left(r\right)$	$\:VIR$
C_60_@PCE	0.016	− 0.011	0.010	− 0.001	0.009
AlC_59_@PCE	0.080	− 0.060	0.092	0.032	0.124
ZnC_59_@PCE	0.056	− 0.071	0.073	0.001	0.074

The QTAIM data (in BCP) clearly support the same interaction-strength trend obtained from the NCI/RDG analysis (Table 7). For C_60_@PCE, the electron density at the BCP is very small ($\:\rho\:\left(r\right)$=0.016), and the associated energy terms are also weak ($\:G\left(r\right)$=0.010 and $\:V\left(r\right)$= -0.001), leading to a small total interaction indicator (Hb = 0.009). This is consistent with the NCI/RDG map, where the interaction region is dominated mainly by green isosurfaces, characteristic of weak van der Waals contacts, and it matches the previously reported weak adsorption behavior for C_60_@PCE. In contrast, AlC_59_@PCE shows the largest BCP electron density (ρ(r) = 0.080), indicating the strongest and most concentrated interaction among the three complexes. It also presents the largest values of the energetic descriptors ($\:G\left(r\right)$=0.092 and $\:V\left(r\right)$=0.032), resulting in the highest Hb value (0.124). This agrees well with the NCI/RDG results, where AlC_59_@PCE exhibited the most pronounced attractive interaction features in the intermolecular region, confirming that PCE binds much more strongly to the Al-doped system. For ZnC_59_@PCE, the BCP properties fall between the other two systems, with ρ(r) = 0.056 and Hb = 0.074, which indicates an interaction stronger than C_60_@PCE but weaker than AlC_59_@PCE. This again overlaps with the NCI/RDG visualization, where ZnC_59_@PCE displayed a mixed pattern of attractive (blue) and van der Waals (green) contributions, suggesting moderate binding strength.

**Fig. 16:**
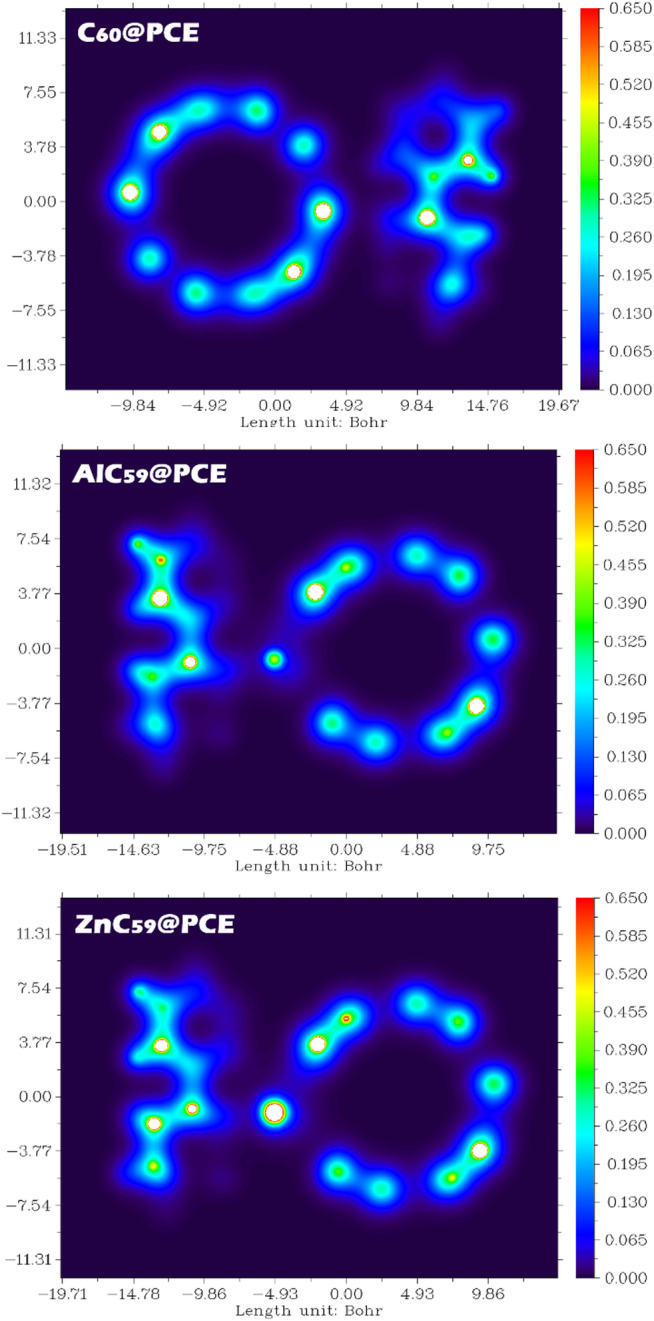
Electron density contour maps for C_60_@PCE, AlC_59_@PCE, and ZnC_59_@PCE, to visualize the electron density distribution in the interaction region.

Electron density maps were studied to visualize the QTAIM findings (See Fig. 16). In these maps, regions of higher electron density (brighter colors) indicate areas of electron accumulation and potential interaction pathways, while the presence or absence of a continuous density region between the interacting fragments provides direct insight into the bonding character. For C_60_@PCE, the electron density is mainly localized on the individual C_60_ cage and the PCE molecule, with only weak and diffuse density appearing in the intermolecular region. The absence of a well-defined, continuous electron density pathway between the nitrogen atom of PCE and the C_60_ surface indicates that no strong bond critical point is formed. This observation is consistent with the low electron density and small energy descriptors at the BCP obtained from QTAIM analysis, confirming that the interaction is dominated by weak van der Waals forces and electrostatic contributions. Such a weak interaction explains the low adsorption energy and the very short recovery time of this system. In contrast, the AlC_59_@PCE electron density map exhibits a pronounced accumulation of electron density in the region between the Al atom and the nitrogen atom of PCE. The formation of a more concentrated and continuous density region clearly indicates the presence of a strong interaction path. This behavior correlates with the high electron density at the bond critical point and the large energy density values reported in the QTAIM results, suggesting a strong and partially covalent (chemisorption-like) interaction. The aluminum dopant acts as an active electron-deficient center, significantly enhancing charge concentration and binding strength with PCE. For ZnC_59_@PCE, the electron density distribution shows an intermediate behavior. An evident increase in electron density is observed in the Zn-N interaction region, confirming dopant-assisted binding; however, the density is less intense and less localized than that observed for AlC_59_@PCE. This indicates a moderately strong interaction, stronger than in pristine C_60_@PCE but weaker than in the Al-doped system. The intermediate BCP electron density and energy values further support this conclusion, reflecting a polarized, predominantly noncovalent interaction.

These findings are fully consistent with the adsorption energy, recovery time, and NCI/RDG/ELF/LOL results, demonstrating that metal doping (particularly with aluminum) significantly enhances electron density localization at the interaction region and strengthens binding with PCE, whereas pristine C_60_ interacts mainly through weak dispersive forces.

### Future work

Although the present study provides a comprehensive theoretical analysis of the interaction between a single PCE molecule and C_60_-based nanostructures, it is important to note that in realistic environments multiple analyte molecules may interact simultaneously or sequentially with the sensor surface. Therefore, future investigations should consider the adsorption of more than one PCE molecule on pristine and metal-doped C_60_ systems. In particular, after the first molecule binds to the primary active site (such as the metal center in AlC_59_ or ZnC_59_), additional molecules may interact with neighboring carbon atoms of the fullerene cage where the interaction strength is expected to be weaker. Studying such multi-adsorption configurations could provide valuable insight into surface coverage effects, cooperative interactions between adsorbates, and possible changes in electronic and sensing properties under higher analyte concentrations. Future theoretical work could therefore explore sequential or simultaneous adsorption of multiple PCE molecules, evaluate how the first adsorption event modifies the electronic structure of the sensor, and determine whether this modification enhances or suppresses the adsorption of additional molecules. Such studies would also allow evaluation of sensor saturation limits, competitive adsorption pathways, and potential changes in conductivity and optical responses at higher molecular coverage. Performing these calculations would require larger computational models and significantly greater computational resources; however, such investigations would provide a more realistic representation of sensing conditions and could further improve the understanding and design of fullerene-based sensing platforms.

## Conclusion

In this work, a comprehensive density functional theory-based investigation was carried out to elucidate, for the first time, the interaction of Eticyclidine (PCE) with pristine C_60_ and metal-doped fullerenes (AlC_59_ and ZnC_59_) for potential sensing and adsorption applications. The reliability of the computational approach was first validated through direct numerical comparison with experimental and literature data. The optimized C-C bond lengths of C_60_ (1.40–1.45 Å) reproduced exactly the experimentally reported range (1.40–1.45 Å), confirming the structural accuracy of the B97D/6-31G* level. Likewise, the calculated HOMO-LUMO gap of pristine C_60_ (1.67 eV) shows very good agreement with experimental values reported in the literature (≈ 1.50–1.86 eV), including 1.85 eV from microwave conductivity measurements and 1.86 eV from high-temperature conductivity studies. The theoretical UV-Vis absorption maximum of isolated C_60_ (360 nm) also closely matches the experimental value of 345 nm, further validating the chosen computational framework.

Metal doping significantly modified the structural and electronic properties of the fullerene cage. Substitution with Al and Zn increased bond lengths to 1.90–1.92 Å (Al-C) and 1.97–2.03 Å (Zn-C), accompanied by marked angular distortions, indicating the formation of highly perturbed and chemically active sites. These distortions were reflected in the electronic descriptors: the energy gap decreased from 1.67 eV (C_60_) to 1.18 eV (AlC_59_) and dramatically to 0.36 eV (ZnC_59_), while chemical softness increased from 0._59_ to 0.84 and 2.77 eV^− 1^, respectively. These numerical trends clearly demonstrate that metal doping substantially enhances fullerene reactivity, in line with previous reports on doped fullerenes used for sensing applications.

Upon interaction with PCE, all systems exhibited notable electronic and optical responses. The interaction distance decreased from 2.80 Å in C_60_@PCE to 1.49 Å in ZnC_59_@PCE and 1.22 Å in AlC_59_@PCE, directly reflecting the increasing interaction strength. This trend was quantitatively supported by adsorption energies of -11.15 kcal.mol^− 1^ (C_60_@PCE), -32.78 kcal.mol^− 1^ (ZnC_59_@PCE), and − 54.08 kcal.mol^− 1^ (AlC_59_@PCE). Correspondingly, recovery times increased dramatically from 1.54 × 10^− 4^ s for C_60_@PCE to 1.09 × 10^12^ s for ZnC_59_@PCE and 4.57 × 10^27^ s for AlC_59_@PCE, clearly distinguishing reversible physisorption on pristine C_60_ from the strong, quasi- chemisorption binding on Al- and Zn-doped cages. These quantitative results were fully consistent with QTAIM parameters, where the electron density at the bond critical point increased from 0.016 (C_60_@PCE) to 0.056 (ZnC_59_@PCE) and 0.080 a.u. (AlC_59_@PCE), and with NCI/RDG, ELF, and LOL analyses, which collectively confirmed weak van der Waals interaction for pristine C_60_ and strong dopant-assisted interactions for metallofullerenes.

From a sensing perspective, pristine C_60_ exhibited the most desirable balance between interaction strength and reversibility. Its moderate adsorption energy (-11.15 kcal.mol^− 1^), ultrafast recovery time, and measurable conductivity change indicate excellent potential for reusable electrochemical detection of PCE. In contrast, AlC_59_ showed exceptionally strong binding, extremely long recovery time, and pronounced charge localization, identifying it as a highly effective adsorbent for PCE capture and removal rather than a reusable sensor. ZnC_59_ displayed intermediate behavior, bridging sensing and adsorption regimes.

Optically, all complexes exhibited pronounced bathochromic shifts upon PCE adsorption. The absorption maximum of C_60_ shifted from 360 to 530 nm, while AlC_59_ and ZnC_59_ showed dramatic red shifts to ~ 765 and ~ 764 nm, respectively, with excitation energies decreasing to ~ 1.61–1.62 eV. These shifts are significantly larger than those of pristine C_60_ and indicate strong electronic coupling with PCE, positioning AlC_59_@PCE in particular as a highly promising colorimetric sensor.

It should be emphasized that the values reported in this work are derived from theoretical calculations and are therefore intended primarily for qualitative comparison and performance evaluation, rather than as exact replacements for experimental measurements. While several calculated parameters show good numerical agreement with available experimental data, the main objective of this study is to provide reliable predictive trends regarding the relative behavior of pristine and metal-doped fullerenes toward PCE. Consequently, these results should be regarded as a theoretical guideline that offers a rational basis for material selection and sensor design, helping to reduce trial-and-error efforts, time, and cost in future experimental investigations rather than substitute for them.

## Data Availability

The data supporting this study are available when reasonably requested from the corresponding author.
